# Coordination of a Neutral Ligand to a Metal Center of Oxohalido Anions: Fact or Fiction?

**DOI:** 10.1021/acs.inorgchem.1c00947

**Published:** 2021-08-05

**Authors:** Anton Kokalj, Žiga Zupanek, Melita Tramšek, Gašper Tavčar

**Affiliations:** † Department of Physical and Organic Chemistry, Jožef Stefan Institute, Jamova 39, 1000 Ljubljana, Slovenia; ‡ Department of Inorganic Chemistry and Technology, Jožef Stefan Institute, Jamova 39, 1000 Ljubljana, Slovenia; § Jožef Stefan International Postgraduate School, Jamova 39, 1000 Ljubljana, Slovenia

## Abstract

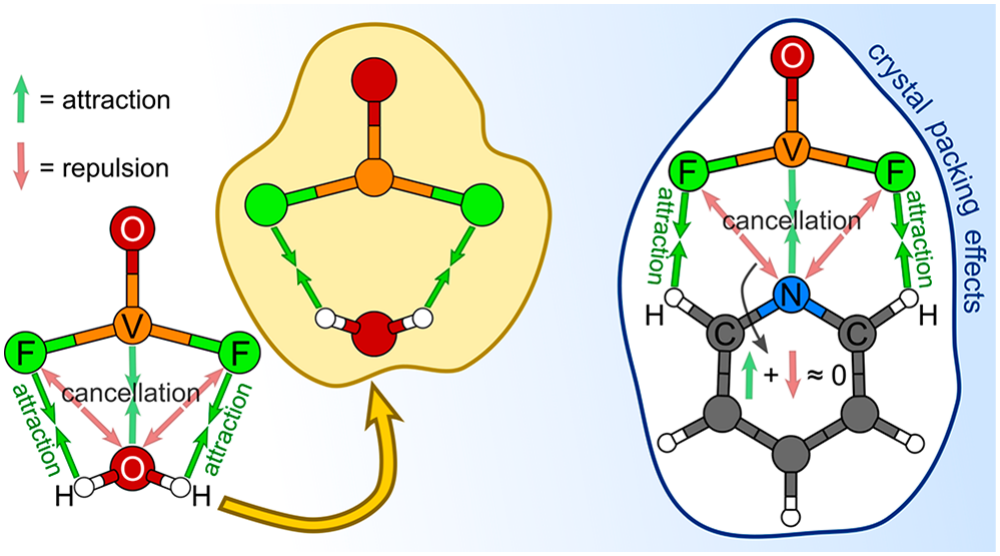

Can a neutral ligand bond to a metal center of a square pyramidal oxohalido anion at the available sixth octahedral position? Crystal structures of some compounds indeed suggest that ligands, such as THF, pyridine, H_2_O, NH_3_, and CH_3_CN, can interact with the central metal atom, because they are oriented with their heteroatom toward the metal center with distances being within the bonding range. However, this assumption that is based on chemical intuition is wrong. In-depth analysis of interactions between ligands and oxohalido anions (e.g., VOX_4_^–^, NbOCl_4_^–^) reveals that the bonding of a neutral ligand is almost entirely due to electrostatic interactions between the H atoms of a ligand and halido atoms of an anion. Furthermore, ab initio calculations indicate that the ligand–VOF_4_^–^ interactions represent only about one-quarter of the total binding of the ligand within the crystal structure, whereas the remaining binding is due to crystal packing effects. The current study therefore shows that relying solely on the structural aspects of solved crystal structures, such as ligand orientation and bond distances, can lead to the wrong interpretation of the chemical bonding.

## Abstract 2

Some crystal structures suggest that ligands interact chemically with the metal center of oxohalido anions. However, analysis shows that the attraction between the heteroatom of the ligand and the metal center is counteracted by the repulsion between the heteroatom and the electronegative F atoms, making electrostatic F···H interactions and crystal packing effects the dominant force holding the ligand and anion together. This is the reason why some ligands rotate with their heteroatom away from the metal center.

## 1 Introduction

The coordination chemistry of anions is a rapidly developing field and has been a subject of several review papers in recent years. The latest review emphasizes the anion–ligand coordination properties with consideration of the structural and geometrical features, where hydrogen bonding dominates the anion coordination behavior; however, anion−π interactions and halogen bonding are also mentioned.^1^ Despite a plethora of reported coordination compounds where anions are coordinated to a metal cation, coordination of a neutral molecule to a metal center in the anionic species is much less common. Such species can be found for the transition metal (V, Nb, Mo, W, Re) square pyramidal oxohalido anions.^2^ Most of them contain either pyridine (Py),^3,4^ tetrahydrofuran (THF),^5−16^ acetonitrile (CH_3_CN),^17−25^ or water^26^ as ligands due to their use as solvents in reactions. The ligand can therefore have access to the sixth coordination site and form a distorted octahedron around the metal center in the anion. However, bonding in such compounds was never studied according to our knowledge. With the current study, we would therefore like to fill this gap and shed light on the ability of the transition metal central atom in such anions to accept an electron-donor molecule. The aim is to resolve the ligand bonding in such anions by determining which interactions contribute to the formation of anion–ligand species (dative bond, hydrogen bond, electrostatic interactions...).

A survey of ligand coordination to a central metal atom of such anions reveals several puzzling aspects. For example, an interesting observation comes from our recent study of the reaction of VOF_3_ with imidazolium fluoride [(L^Dipp^)H][F] (L^Dipp^ = 1,3-bis(2,6-diisopropylphenyl)imidazol-2-ylidene) that forms discrete [VOF_4_]^−^ anions.^27^ Crystallization of the product in acetonitrile yielded solvate crystals [(L^Dipp^)H][VOF_4_]·2CH_3_CN where nitrogen atoms of acetonitrile molecules are turned away from the vanadium metal center. This is puzzling because compounds with acetonitrile coordinated with nitrogen atoms to the metal center are known for closely related [NbOCl_4_]^−^ and [NbOBr_4_]^−^ anions,^18,20,23−25^ although crystal structures of [VOCl_4_]^2–^ compounds show incorporated acetonitrile solvent molecules, similarly to our example, as not being coordinated to the metal center.^28,29^ Moreover, electronic structure analysis shows that the vanadium atom is more positively charged in [VOF_4_]^−^ (+2.3) than in [VOCl_4_]^−^ (+1.9), and as such the former should be a better ligand acceptor.^27^ In contrast, crystal structures of [NbOF_4_]^−^^30^ and [VOF_4_]^−^^31,32^ with water molecules coordinated to the metal center are known. A comparison of acetonitrile and water molecules as ligands in these coordination systems shows that water has, in addition to the electron-rich heteroatom capable of coordination, positively charged hydrogen atoms that can form hydrogen bonds with the anions, whereas no such positively charged hydrogen atoms are present in acetonitrile. Why is it then that in the above quoted compounds the water molecule is coordinated with its oxygen atom toward the central metal atom, whereas acetonitrile is oriented with the methyl group toward the metal center? For the reasons described above, we have therefore decided to study such anion–ligand interactions in detail in order to shed some light on the bonding, stability, and geometry of such compounds.

We will show with several examples that a neutral ligand is not attached to the anion via the bonding of its electronegative heteroatom to the metal center but instead by the weak F···H–C electrostatic interactions and to even larger extent by the crystal packing effects. With this being so, is it therefore appropriate to speak about the heteroatom–metal bond? Most likely not, and this holds true even in the context a very broad definition of a chemical bond put forward by Linus Pauling: “*there is a chemical bond between two atoms or two groups of atoms in case that the forces acting between them are such as to lead to the formation of an aggregate with sufficient stability to make it convenient for the chemist to consider it as an independent chemical species.”*^33^

An interesting account on the concept of chemical bonding was made by Dunitz and Gavezzotti for the case of intermolecular interactions.^34^ In particular, they posed the following questions: “*What, then, should one do about distinguishing genuine intermolecular bonds from indiscriminate atom–atom contacts? Where should one stop talking and thinking about bonds? At a certain threshold distance? At a certain threshold energy?*” We will show herein that these questions are relevant not only for the intermolecular bonding, but also for the intramolecular bonding, where the term “intramolecular” implies distances between chemical species that are so much smaller than the respective sum of the van der Waals radii as to approach the sum of the respective covalent (or cationic/anionic) radii. On the basis of chemical intuition, such distances would *de facto* qualify as “chemical bonding.” But if the interaction energy between the heteroatom of a ligand and the oxohalido anion is vanishing or even repulsive, as will be shown herein, then it seems that the use of the term “chemical bond” is misleading, despite the short contact distance. The question posed by Dunitz and Gavezzotti can therefore be generalized by dropping the adjective “intermolecular” as to include also intramolecular contacts. The question so amended then reads, “*What, then, should one do about distinguishing genuine bonds from indiscriminate atom–atom contacts?*”

## 2 Experimental Section

All experiments were carried out under an inert atmosphere of dry argon using standard Schlenk and glovebox techniques. Use of reaction vessels made of polymers is advised as HF starts to form with slow decomposition of reactants. Starting reagents [(L^Dipp^)H][F]^35^ and [(L^Dipp^)H][VOF_4_]^27^ were prepared according to the synthetic procedure in the literature. VOF_3_ (99%) was obtained from Alfa Aesar and used as received. Tetrahydrofuran (THF; 99.8%) obtained from Merck was dried with sodium and benzophenone until the solution turned deep purple, was distilled under inert conditions, freeze–thawed, and was stored in glovebox over 3 Å molecular sieves for at least 48 h prior to use. Pyridine (Py) (99.8%) obtained from Acros Organics was stored in a glovebox and dried over 3 Å molecular sieves.^27^ Dichloromethane (DCM) was distilled under inert conditions, freeze–thawed, and stored over 3 Å molecular sieves.

### 2.1 Synthesis Procedure

#### 2.1.1 Synthesis of [(L^Dipp^)H][VOF_4_(THF)]

A solution of [(L^Dipp^)H][F] (396 mg; 0.969 mmol) in 10 mL of THF was added to the solution of VOF_3_ (120 mg; 0.968 mmol) in 5 mL of THF in a FEP (fluorinated ethylene propylene) container. Crystals suitable for X-ray structural analysis formed with slow evaporation of the solvent. The reaction most likely has quantitative yield, but when the crystals are removed from the mother liquor, they begin to decompose rapidly, and the diffraction patterns of such crystals become poor, most likely due to the evaporation of the THF compound and the resulting degradation of the overall crystal structure. For this reason, it was not possible to quantitatively determine the yield.

#### 2.1.2 Synthesis of [(L^Dipp^)H][VOF_4_(Py)]

[(L^Dipp^)H][VOF_4_] (400 mg; 0.751 mmol) was dissolved in a FEP (fluorinated ethylene propylene) container with the addition of 10 mL of DCM. Afterward 1 equiv of Py (60 mg; 0.759 mmol) was added to the reaction mixture. Within a few days, crystals suitable for X-ray structural analysis formed from a concentrated solution. The yield is most likely quantitative, but the product begins to slowly decompose after removal from the mother liquor.

### 2.2 Crystal X-ray Structural Analysis

Details of the crystallographic data collection and refinement parameters are given in Table S1 in the Supporting Information (CCDC deposition numbers for [(L^Dipp^)H][VOF_4_(THF)] (**1**), 2065280; [(L^Dipp^)H][VOF_4_(Py)] (**2**), 2065279). For the collection of crystal data, graphite monochromated Cu Kα radiation was used on a Gemini A diffractometer equipped with an Atlas CCD detector. Crystals were held at 150 K with a stream of a nitrogen gas. The data were treated using the CrysAlisPro software suite program package.^36^ Analytical absorption correction was applied to all data sets.^37^ Structures were solved with SHELXT^38^ and structure refinement performed with the SHELXL,^39^ both implemented in the program package Olex2.^40^

### 2.3 Computational Details

#### 2.3.1 DFT Calculations of Crystal Structures

Calculations of crystal structures were performed in the framework of density functional theory (DFT), using the generalized gradient approximation of Perdew–Burke–Ernzerhof^41^ supplemented with the D3 empirical dispersion correction of Grimme with Becke–Johnson (BJ) damping,^42,43^ labeled as PBE-D3. We used the plane-wave method with ultrasoft pseudopotentials^44,45^ as implemented in the PWscf code from the Quantum ESPRESSO distribution.^46^ Kohn–Sham orbitals were expanded in a plane-wave basis set up to a kinetic energy cutoff of 50 Ry (400 Ry for the charge-density cutoff); these cutoffs yield well converged results. All degrees of freedom including the unit cell size and shape were relaxed. Brillouin-zone integrations were performed using only a gamma k-point.

We also made some calculations of standalone complexes using the “*molecule in a box*” approach with a large cubic box of 25 Å size and Makov–Payne correction.^47^ Bader charge analyses were performed using the Bader code^48,49^ by generating charge densities with the PAW (projector-augmented-wave) potentials^50^ and 1000 Ry kinetic energy cutoff for charge density.

#### 2.3.2 Molecular Calculations of Isolated Complexes, Molecules, and Ions

Molecular calculations were also performed with the Gaussian16 program^51^ using three different methods. For consistency with the crystal structure calculations described above, we used the PBE-D3 functional with BJ damping. In addition, we also used Grimme’s double-hybrid B2PLYP functional^52^ combined with D3-BJ dispersion correction, labeled as B2PLYPD3. Some benchmark calculations were also performed with a CCSD method.^53^ Electrons were described with all electron def2TZVP basis sets.^54^ Basis-set-superposition errors (BSSE) were estimated using the Boys–Bernardi counterpoise correction.^55^

Molecular graphics were produced by the XCRYSDEN graphical package.^56^

#### 2.3.3 Energy and Density Equations

The binding between ligand L and the VOF_4_^–^ anion in the standalone [VOF_4_(L)]^−^ complex was estimated as
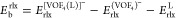
1where “rlx” stands for relaxed and *E*_rlx_ designates total (potential) energies of a relaxed structures, i.e., the [VOF_4_(L)]^−^ complex and VOF_4_^–^ and L constituents. A rigid or gross binding energy (*E*_b_^rigid^) is calculated similarly, but with the geometries of VOF_4_^–^ and L kept the same as in the complex:
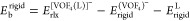
2where *E*_rigid_ stands for total (potential) energies of “rigid” VOF_4_^–^ and L constituents.

The gross binding of a single ligand within the crystal structure is calculated as
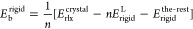
3where *n* is the number of ligands L in the unit cell, *E*_rlx_^crystal^ is the total (potential) energy of the crystal structure per unit cell, *E*_rigid_^L^ is the total energy of the standalone ligand in the unit cell, and *E*_rigid_^the-rest^ is the total energy of “the rest”, where the meaning of “the rest” is such that “*n*L + the-rest = whole crystal.” The rigid binding energy between a single ligand and a single VOF_4_^–^ ion in the crystal structure is calculated by extracting a single [VOF_4_(L)]^−^ complex from the crystal structure and calculating
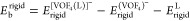
4where the *E*_rigid_^[VOF_4_(L)]^−^^ is the total energy of a [VOF_4_(L)]^−^ complex having the same geometry as in the crystal structure. Beware that the rigid binding energies, calculated with eqs 2–4, are designated with the same label *E*_b_^rigid^, but it will be always specifically indicated to which equation a given reported *E*_b_^rigid^ corresponds.

Intermolecular interactions in the crystal structure are visualized by means of the electron charge density difference, calculated as
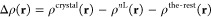
5where the superscripts “crystal,” “*n*L,” and “the-rest” have analogous meanings to those in eq 3, i.e., “*n*L + the-rest = whole crystal”. Note that the “*n*L” and “the-rest” structures are kept the same as in the whole crystal structure.

The interaction between a ligand L and the VOF_4_^–^ anion in the [VOF_4_(L)]^−^ complex was also scrutinized by molecular orbital analysis, and to this end we utilized a density of states (DOS) like approach, where discrete molecular states were broadened by a Gaussian smearing. In particular, the ligand–anion interaction was analyzed by DOS of the [VOF_4_(L)]^−^ complex projected onto individual molecular orbitals of either a ligand L or the VOF_4_^–^ anion, i.e.,
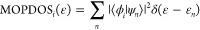
6where MOPDOS stands for Molecular-Orbital Projected DOS, ψ_*n*_ and ε_*n*_ are molecular orbitals of the [VOF_4_(L)]^−^ complex and their eigenvalues, respectively, ϕ_*i*_ is a particular molecular orbital of either L or the VOF_4_^–^ fragment, and the Dirac δ function is approximated by a Gaussian function with the smearing parameter of 0.04 eV. MOPDOSes were calculated using the molecularpdos.x utility^57^ of Quantum ESPRESSO.

Although the kJ/mol energy unit is used herein for binding energies, the energy unit of eV is used instead for molecular orbital eigenvalues and DOS plots. While the use of two energy units may seem inconsistent, the choice can be justified by the observation that DOS analyses are frequently performed with the eV energy unit, whereas the unit of kJ/mol is more common for bond energies.

## 3 Results and Discussion

### 3.1 Experimental Results

#### 3.1.1 Synthesis of Compounds

Reactions between [(L^Dipp^)H][VOF_4_] salt and certain ligands were performed under inert conditions. The premise of the study was that electron donating ligands are able to interact with the negatively charged [VOF_4_]^−^ moiety. Although no stable adducts were isolated in powder form, crystals of [(L^Dipp^)H][VOF_4_(THF)] (**1**) and [(L^Dipp^)H][VOF_4_(Py)] (**2**) were obtained. Other similar ligands, i.e., 1,4-dioxane, benzophenone, piperidine, phenazine, 2,2′-bipyridine, and 1,10-phenanthroline, were also tested; however, no such adducts were detected. Previously, we already reported a solvate crystal structure of [(L^Dipp^)H][VOF_4_]·2CH_3_CN, where acetonitrile does not interact with the vanadium atom of the anion.^27^

#### 3.1.2 Crystal Structure of [(L^Dipp^)H][VOF_4_(THF)] (**1**)

**Figure 1:**
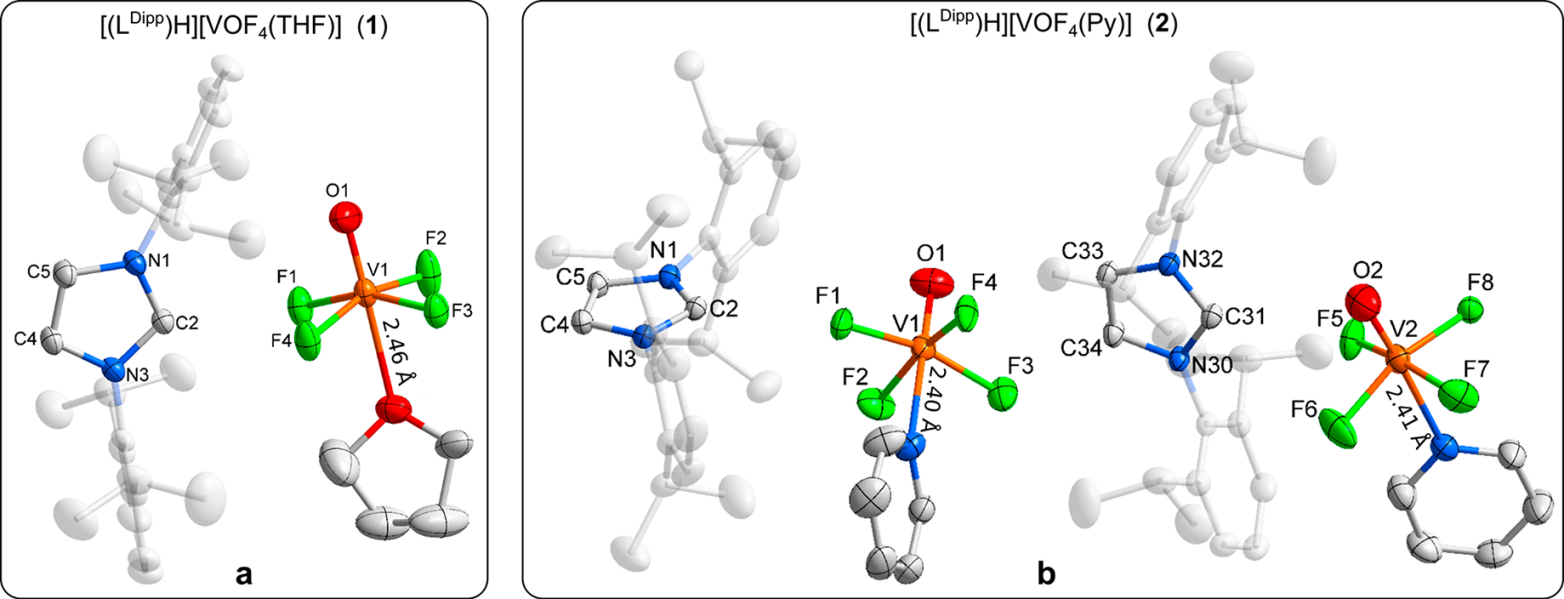
Images of experimentally determined crystal structures. (a) Asymmetric unit of the [(L^Dipp^)H][VOF_4_(THF)] (**1**). For clarity reasons, only domain B of the THF molecule is shown, and the respective V–O_THF_ bond length between the THF ligand and the vanadium center is stated. (b) Asymmetric unit of the [(L^Dipp^)H][VOF_4_(Py)] (**2**). The V–N_Py_ bond lengths are also given. In both images ellipsoids are drawn at 50% probability, and for clarity reasons “wingtips” of [(L^Dipp^)H]^+^ are shaded and hydrogen atoms are omitted.

Crystallization of [(L^Dipp^)H][VOF_4_] in tetrahydrofuran (THF) led to the isolation of [(L^Dipp^)H][VOF_4_(THF)] (**1**) crystals. The corresponding asymmetric unit is shown in Figure 1a, and crystal structure data are presented in the Supporting Information (Tables S1 and S2). Crystal structure analysis showed the THF molecule situated on the sixth coordination position of the vanadium(V) coordination sphere. Crystals taken from the concentrated solution for crystallization presumably lose weakly bound THF molecules, resulting in a noticeably weaker diffraction pattern. Further examination of the crystal structure of the [(L^Dipp^)H][VOF_4_(THF)] (**1**) complex showed the V=O bond to be of the same length as in the disordered [VOF_4_]^−^ anion, whereas the V–F bonds are marginally longer in the [VOF_4_(THF)]^−^ anion (Table S4). The THF molecule occupies two positions that are populated almost equivalently (domain A, 46% and B, 54% at *T* = 150 K). The V–O_THF_ bond length is 2.40 Å in domain A and 2.46 Å in domain B, thus being considerably shorter than the respective sum of the van der Waals radii (3.92 Å).^58^ The comparison of the V–O_ligand_ distances in the [VOF_4_(THF)]^−^ anion to the structurally related water containing [VOF_4_(H_2_O)]^−^ anions^31,32^ reveals that V–O_THF_ (about 2.4 Å) is notably longer than V–O_H_2_O_ (about 2.3 Å) most likely due to the larger size of the THF molecule. Fluorine atoms of the [VOF_4_(THF)]^−^ anion are pushed toward the oxygen so that the O–V–F angles decrease in comparison to the starting [VOF_4_]^−^ anion (Table S4). Several other selected examples of structurally characterized compounds with oxofluorido anions are presented in the Supporting Information (Table S5).

#### 3.1.3 Crystal Structure of [(L^Dipp^)H][VOF_4_(Py)] (**2**)

Starting salt [(L^Dipp^)H][VOF_4_] readily dissolves when pyridine (Py) is used as a solvent. Slow evaporation of the solvent yields only a few crystals of [(L^Dipp^)H][VOF_4_(Py)] (**2**). Crystals of much better quality were obtained when [(L^Dipp^)H][VOF_4_] and Py in equimolar ratio were dissolved in dichloromethane (DCM) and the reaction mixture was slowly concentrated. Crystal structure analysis determined that the asymmetric unit comprises two subunits. The asymmetric unit is presented in Figure 1b, and crystal structure data are presented in the Supporting Information (Tables S1 and S3). The V=O bond is in both subunits insignificantly elongated in comparison to the same bond in the [VOF_4_]^−^ anion, and V–F bond lengths are comparable to those in the [VOF_4_(THF)] anion (Table S4). The V–N_Py_ bond length of 2.40 Å is considerably shorter than the sum of the respective van der Waals radii (4.08 Å).^58^ With the introduction of Py to the coordination sphere, the decrease of the O–V–F angles is even more prominent than with the THF molecule.

A further comparison of structural data between [VOF_4_]^−^, [VOF_4_(THF)]^−^, and [VOF_4_(Py)]^−^ is presented in the Supporting Information (Tables S4 and S5). Herein, we only commented the most revealing structural features in both compounds that indicate coordination of the solvent molecule to the metal center due to vicinity of the heteroatom of the ligand pointing toward the vanadium by slight elongation of bonds associated with vanadium(V) center and reduction of the O–V–F angles.

### 3.2 Computational Results

The analysis of the type of bonding between the [VOF_4_]^−^ anion and neutral molecules (THF and Py) was performed by means of molecular modeling and is presented below. To start with, periodic PBE-D3/plane-wave calculations reproduce the crystal structures of [(L^Dipp^)H][VOF_4_(THF)] (**1**) and [(L^Dipp^)H][VOF_4_(Py)] (**2**) compounds in very good agreement with experimental results (cf. Table S6 in the Supporting Information). In addition to the periodic PBE-D3/plane-wave crystal structure calculations, we also performed relaxation calculations of isolated [VOF_4_(L)]^−^ units, because such calculations allow the use of more sophisticated methods; in particular, we used the double-hybrid dispersion corrected B2PLYPD3 functional and the CCSD method.

#### 3.2.1 Calculated Structure of [VOF_4_(THF)]^−^

**Figure 2:**
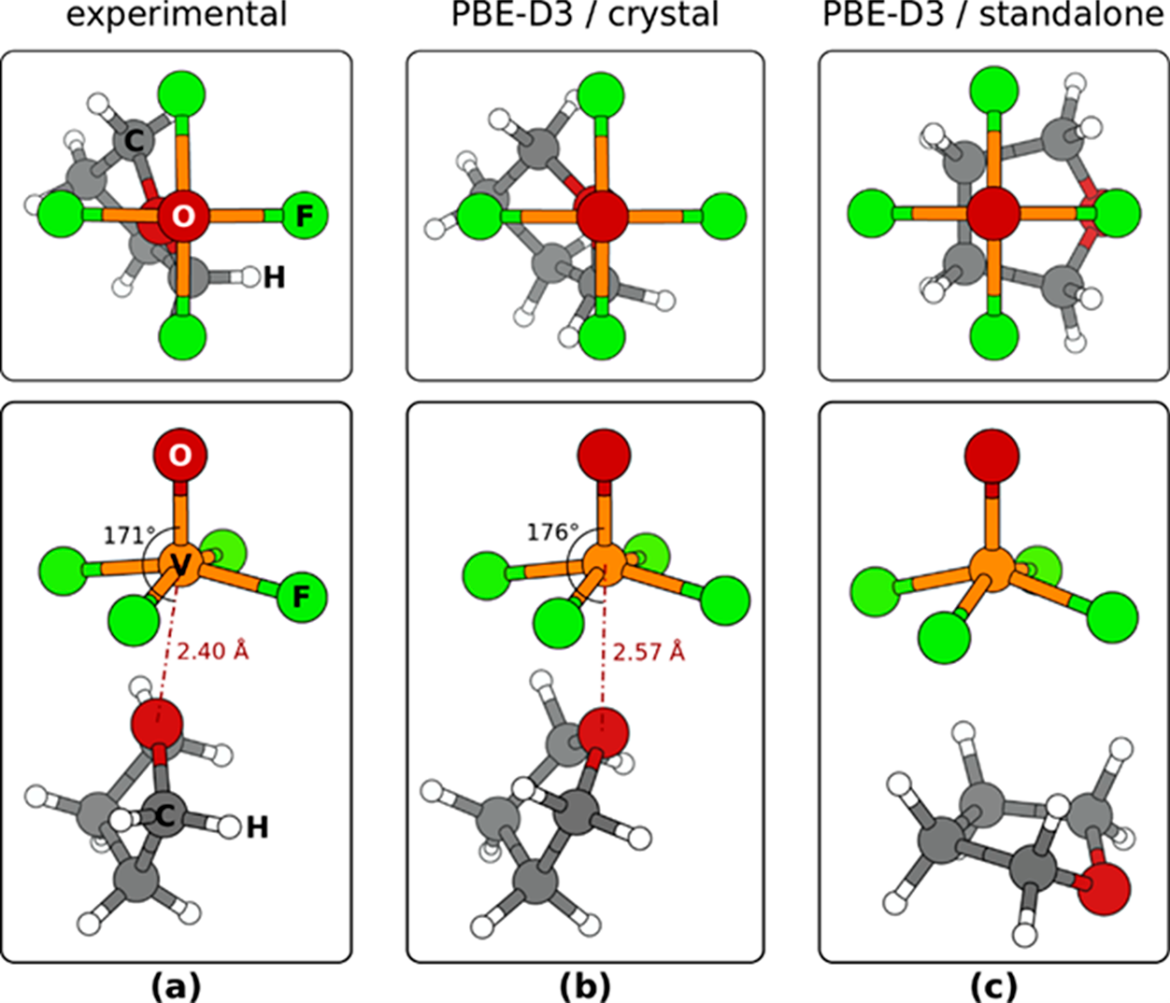
Top- and perspective-view snapshots of the [VOF_4_(THF)]^−^ unit in the (a) experimentally determined and (b) PBE-D3/plane-wave calculated crystal structure of [(L^Dipp^)H][VOF_4_(THF)] (**1**). (c) Snapshots of calculated, isolated [VOF_4_(THF)]^−^ unit. For clarity, surrounding atoms in the crystal structure are not shown. Note that in the crystal structure the THF is oriented “vertically” with its O_THF_ atom toward the V atom, whereas in the standalone unit the THF is oriented close to horizontal such that the four “upper” H atoms of the molecule point toward the equatorial F atoms and the O_THF_ atom points away from the V atom. In the crystal structure THF is in the twisted conformation, whereas in the standalone unit it shows the envelope conformation.

A notable finding, resulting from our calculations, is that while the PBE-D3/plane-wave calculation of the [(L^Dipp^)H][VOF_4_(THF)] (**1**) crystal structure reproduces the geometry of the [VOF_4_(THF)]^−^ unit in fair agreement with the experimental results, the calculations of the isolated unit give a completely different structure (Figure 2). In particular, in the crystal structure, the THF molecule is located at the sixth octahedral coordination site of the V(+5) metal center and oriented with its O_THF_ atom toward the V atom, such that the O=V···O_THF_ angle is close to 180° (expt. 171° and calcd. 176°, Figure 2a,b); this geometry will be referred to as “vertical.” In contrast, in the calculated isolated unit, the THF molecule rotates such that its ring is close to parallel to the plane of equatorial F atoms or close to normal to the V=O bond (Figure 2c); this geometry will be referred to as “horizontal.” In this geometry, the four “upper” H atoms of the THF molecule point toward the equatorial F atoms such that each H forms a bifurcated F···H–C “bond” with two F atoms (Figure 2c). Such “horizontal” geometry of THF in the isolated unit is predicted not only with PBE-D3 but also with B2PLYPD3 and CCSD calculations (cf. Table 1).

**Table 1:** Binding Energies (*E*_b_^rlx^), eq 1, between VOF_4_^–^ Anion and THF, Py, or ABCO (1-Azabicyclo[2.2.2]octane) in the Standalone [VOF_4_(THF)]^−^, [VOF_4_(Py)]^−^, or [VOF_4_(ABCO)]^−^ Complexes, Respectively, and between the VOCl_4_^–^ or NbOCl_4_^–^ Anion and CH_3_CN in the Standalone [VOCl_4_(CH_3_CN)]^−^ and [NbOCl_4_(CH_3_CN)]^−^ Complexes^a^

	isolated [VOF_4_–THF]^−^					
	“vertical”-THF	“horizontal”-THF	isolated [VOF_4_–Py]^−^	isolated [VOF_4_–ABCO]^−^	isolated [VOCl_4_–CH_3_CN]^−^	isolated [NbOCl_4_–CH_3_CN]^−^
PBE-D3/def2TZVP						
*E*_b_^rlx^ (kJ/mol)	–18^b^ (−11^c^)	–39 (−32)^c^	–18 (−10^c^)	–36 (−25^c^)	+14^b^ (+20^c^)	–2^b^ (+2^c^)
*d*_M···X-ligand_ (Å)	3.02^b^	4.77	2.64	2.73	2.53^b^	2.60^b^
∠O=M···X_ligand_ (deg)	constrained^b^		180	178	constrained^b^	constrained^b^
B2PLYPD3/def2TZVP						
*E*_b_^rlx^ (kJ/mol)	–26 (−15^c^)	–38 (−31^c^)	–29 (−18^c^)	–50 (−35^c^)	+3^b^ (+11^c^)	–9^b^ (−3^c^)
*d*_M···X-ligand_ (Å)	2.62	4.70	2.51	2.58	2.40^b^	2.58^b^
∠O=M···X_ligand_ (deg)	178		180	177	constrained^b^	constrained^b^
CCSD/def2TZVP						
*E*_b_^rlx^ (kJ/mol)	–21 (−5^c^)	–33 (−25^c^)				
*d*_M···X-ligand_ (Å)	2.69	5.28				
∠O=M···X_ligand_ (deg)	177					

a Binding energies in parentheses are corrected for BSSE. M···X_ligand_ bond lengths (*d*_M···X-ligand_) and O=M···X_ligand_ bond angles (∠O=M···X_ligand_) are also given, where M = V or Nb and X_ligand_ = O_THF_, N_py_, N_ABCO_, or N_CH_3_CN_.

b O=M···X_ligand_ angle constrained to 180°.

c BSSE corrected value with the counterpoise correction.

The geometry of THF itself also differs in the [(L^Dipp^)H][VOF_4_(THF)] crystal and in the standalone [VOF_4_(THF)]^−^ unit, because within the crystal (for both experimentally determined and calculated structures), THF displays the twisted conformation, whereas in the standalone unit (calculated structure), it shows the envelope conformation (cf. Figure 2a,b vs c). However, it should be noted that the energy difference between the two isolated THF conformers is below 1 kJ/mol (PBE-D3 predicts that the envelope conformer is more stable by 0.2 kJ/mol, whereas B2PLYPD3 and CCSD predict the twisted structure to be more stable by 0.4 and 0.3 kJ/mol, respectively; for a more thorough discussion on the stability of the two conformers see, e.g., ref (59)). Such a small energy difference between the two THF conformers indicates that the preference among them can be easily driven by intermolecular interactions.

The V···O_THF_ distance is also affected significantly by intermolecular interactions (i.e., crystal packing effects). In particular, the PBE-D3 calculated V···O_THF_ distance in the standalone “vertical” structure of the [VOF_4_(THF)]^−^ complex is 3.02 Å (obtained with constrained relaxation), which is considerably longer than the experimental value of 2.40 Å; even the respective B2PLYPD3 (2.62 Å) and CCSD (2.69 Å) distances are significantly overestimated (cf. Table 1). This overestimation is clearly due to the absence of crystal packing effects in the standalone complex, because PBE-D3 calculations predict that the V···O_THF_ distance is reduced from 3.02 Å in the standalone “vertical” complex (Table 1) to 2.57 Å in the crystal structure (Figure 2c).

#### 3.2.2 Calculated Structure of [VOF_4_(Py)]^−^

**Figure 3:**
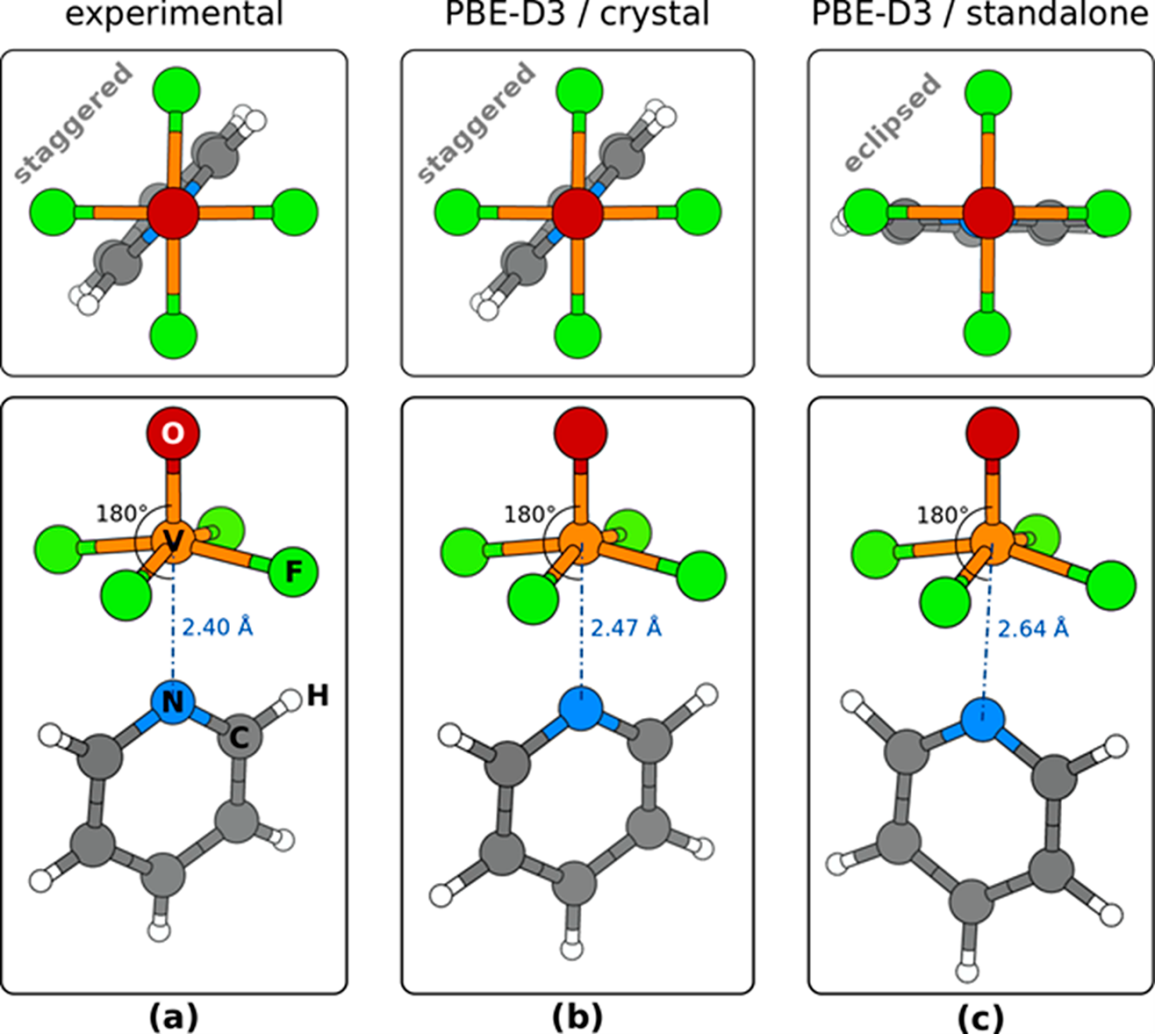
Top- and perspective-view snapshots of the [VOF_4_(Py)]^−^ unit in the (a) experimentally determined and (b) PBE-D3/plane-wave calculated crystal structure of [(L^Dipp^)H][VOF_4_(Py)] (**2**). (c) Snapshots of the calculated isolated [VOF_4_(Py)]^−^ unit. For clarity, surrounding atoms in the crystal structure are not shown. The top-view snapshots reveal that in the crystal structure the plane of the Py molecule is staggered with respect to equatorial F atoms, whereas in the standalone unit it is eclipsed. Beware that in the crystal structure there are two symmetry nonequivalent [VOF_4_(Py)]^−^ units; one displays an O=V···N_Py_ angle of 180° (shown here) and the other 177° (not shown).

In contrast to the previous case of THF, the Py molecule is oriented with its N_Py_ atom toward the V cation both in the crystal structure and in the isolated [VOF_4_(Py)]^−^ unit (Figure 3). While for THF the number of F···H interactions are maximized in the “horizontal” geometry, the geometry of the Py molecule is planar and the F···H interactions are optimal in the “vertical” geometry, where two positively charged H atoms point toward negatively charged F atoms, whereas in the “horizontal” geometry the positively charged H atoms would point away from negatively charged F atoms. Still, there is a difference between the geometry of the [VOF_4_(Py)]^−^ unit in the crystal structure and in the isolated structure. In particular, in the crystal structure the molecular plane of Py is staggered with respect to F atoms, whereas in the isolated unit the Py is predicted (by PBE-D3 and B2PLYPD3 calculations) to be eclipsed with two F atoms (see the top-view snapshots in Figure 3).

Like for the aforementioned V···O_THF_ distance, also here the crystal packing effects have a significant effect on the V···N_Py_ distance; the PBE-D3 predicted value for the standalone complex is 2.64 Å (B2LYPD3 gives 2.51 Å), whereas in the crystal the PBE-D3 calculated distance reduces to 2.47 Å (experimentally 2.40 Å).

#### 3.2.3 Analysis of the L···VOF_4_ Bonding (L = THF and Py)

**Figure 4:**
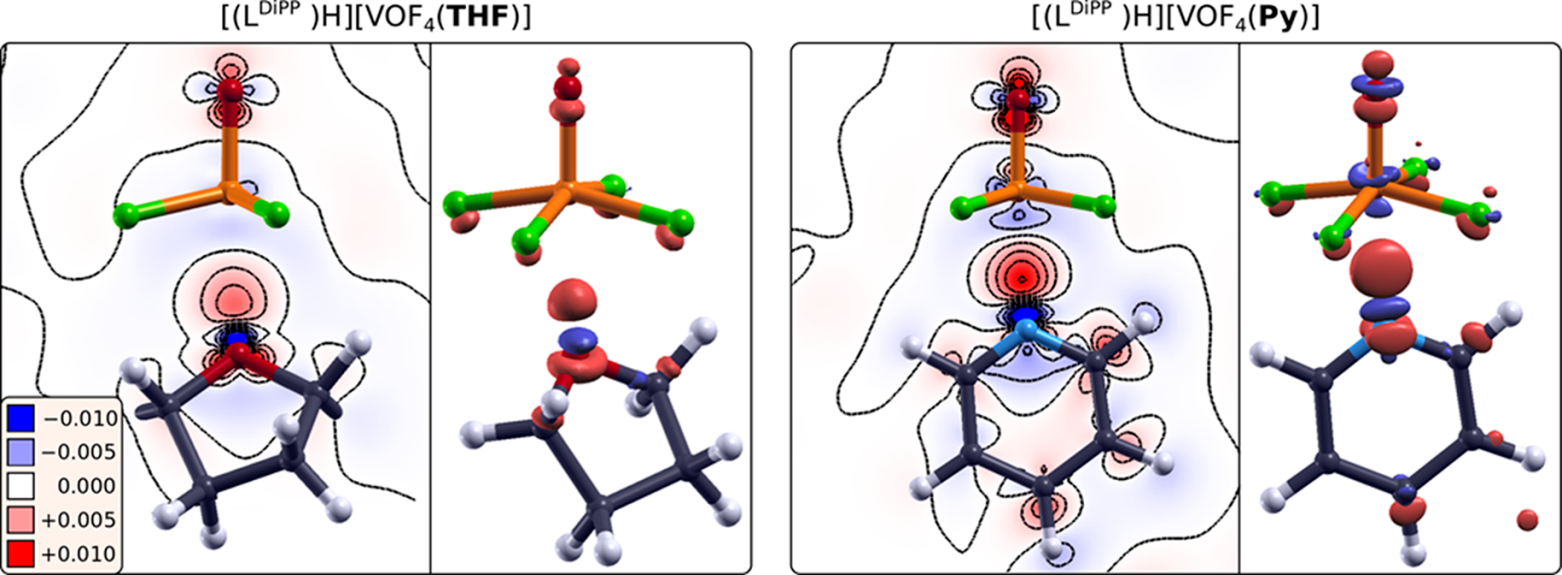
Electron density difference plots of [(L^Dipp^)H][VOF_4_(L)] for L = THF (left) and Py (right), calculated with eq 5. The charge redistribution is the largest around the [VOF_4_(L)]^−^ units, which are in the focus. For clarity, surrounding atoms in the crystal structure are not shown. Electron excess regions are colored red, and electron deficit regions are blue, i.e., electrons flow from blue to red regions. Isosurfaces are drawn at ±0.004 e/Bohr^3^, and contour plots are plotted along the V···L bonding plane in linear scale from −0.01 to +0.01 e/Bohr^3^ with an increment of 0.002 e/Bohr^3^. Note that charge redistribution is considerably larger for Py and is more akin to covalent bonding. For [VOF_4_(Py)]^−^, only one of the two symmetry nonequivalent units is shown, i.e., the one with the O=V···N_Py_ angle of 177°. These plots demonstrate that the L molecules are charge-neutral in the [VOF_4_(L)]^−^, because the electron transfer between VOF_4_^–^ and L is rather minor and mostly confined to the V···L bonding region. According to Bader population analysis, the charges of THF and Py within the [VOF_4_(L)]^−^ are +0.007 and +0.036, respectively.

The fact that calculations predict a “vertical” THF geometry in the crystal structure and a “horizontal” one for the standalone [VOF_4_(THF)]^−^ suggests that the “vertical” geometry in the crystal structure is due to crystal packing effects. This inference is strongly supported by the analysis of the binding of THF within the crystal structure (Table 2), which reveals that the interaction of THF with VOF_4_^–^ represents only about 20% of the total binding, whereas the remaining 80% comes from the crystal packing effects that are mainly due to London dispersion interactions (the ratio for Py and VOF_4_^–^ is similar, 24% vs 76%). The electron density difference plots (Figure 4) suggest that the V···N_Py_ interaction within [VOF_4_(Py)]^−^ is stronger and more akin to covalent bonding than the V···O_THF_ interaction of “vertical” THF in the crystal structure, which is consistent with the tabulated rigid binding energies of Py and “vertical” THF with VOF_4_^–^ in the crystal structure (Table 2). The calculated binding energies clearly reveal that Py is more strongly bound than THF within the crystal structure (Table 2), which is consistent with the observed greater stability of Py-containing single crystals [(L^Dipp^)H][VOF_4_(Py)] (**2**) compared to that of THF crystals [(L^Dipp^)H][VOF_4_(THF)] (**1**). Crystallinity of the latter much more rapidly deteriorates when withdrawn from the mother liquor, and the compound decomposes if exposed to air.

**Table 2:** Rigid Binding Energies (*E*_b_^rigid^), eq 3, of THF, Py, H_2_O, and CH_3_CN Molecules within the Crystal Structures of [(L^Dipp^)H][VOF_4_(THF)] (**1**), [(L^Dipp^)H][VOF_4_(Py)] (**2**), Hypothetical [(L^Dipp^)H][VOF_4_(H_2_O)], Hypothetical [L^PPh3Me^][VOCl_4_(CH_3_CN)], and [L^PPh3Me^][NbOCl_4_(CH_3_CN)] (Labeled As Environment = Crystal)^a^

		*E*_b_^rigid^ (kJ/mol)	
compound	environment	PBE-D3/plane-wave	PBE-D3/def2TZVP
[VOF_4_(THF)]^−^	crystal	–85	
	standalone	–17	–23 (−14^b^)
[VOF_4_(Py)]^−^	crystal	–110	
	standalone	–26	–31 (−22^b^)
[VOF_4_(H_2_O)]^−^	crystal	–42	
	standalone	–28	–35 (−26^b^)
[VOCl_4_(CH_3_CN)]^−^	crystal	–97	
	standalone	–4	–3 (+4^b^)
[NbOCl_4_(CH_3_CN)]^−^	crystal	–111	
	standalone	–15	–14 (−10^b^)

a For comparison, the rigid binding energies between L and MOX_4_^–^ (L = THF, Py, H_2_O, CH_3_CN; M = V, Nb; and X = F, Cl) in the standalone complexes are also given (labeled as environment = standalone); they were calculated with eq 4 for standalone [MOX_4_(L)]^−^ structures having the same geometry as in the respective crystal structure. Please note the conformity of PBE-D3/plane-wave and PBE-D3/def2TZVP results for standalone complexes; plane-wave basis set is not subject to BSSE but def2TZVP is, hence the BSSE uncorrected def2TZVP values are usually more exothermic and BSSE corrected values (stated in parentheses) are usually slightly less exothermic than the plane-wave results, as one would expect.

b BSSE corrected value with the counterpoise correction.

**Figure 5:**
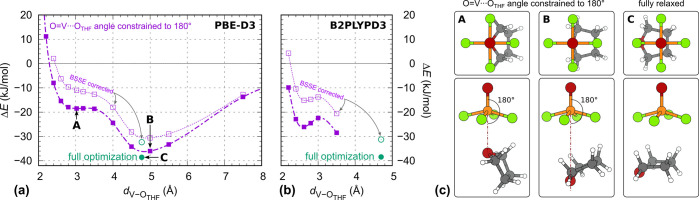
(a) PBE-D3/def2TZVP and (b) B2PLYPD3/def2TZVP calculated interaction energy for isolated [VOF_4_(THF)]^−^ as a function of the V···O_THF_ distance with the O=V···O_THF_ angle constrained to 180° (dashed curves represent BSSE corrected energies). Zero energy is set to the sum of energies of isolated relaxed THF and VOF_4_^–^. (c) Structures corresponding to the points labeled A, B, and C. In the structure A, THF is oriented “vertically” with O_THF_ facing toward the V cation. PBE-D3 predicts that this point corresponds to a wide plateau, while B2PLYPD3 gives a shallow minimum. Upon elongation of the V···O_THF_ distance, THF rotates from “vertical” to “horizontal” orientation and reaches a more stable state B at a distance of about 5 Å. Upon releasing the O=V···O_THF_ constraint, the THF molecule displaces mainly “horizontally” as to optimize the F···H interactions and reaches presumably a global minimum (structure C).

To understand the interplay between the “vertical” and “horizontal” orientations of THF within the standalone [VOF_4_(THF)]^−^, we performed a series of constrained optimizations with PBE-D3 and B2PLYPD3 functionals for the standalone complex, where the O=V···O_THF_ angle of the initial “vertical” structure was constrained to 180° and the V···O_THF_ distance was stepwise increased. Figure 5 shows the resulting interaction energies as a function of the V···O_THF_ distance. The presented energy profiles show that for PBE-D3 there is a flat plateau around the V···O_THF_ distance of 3.0 Å (structure A in Figure 5), whereas B2PLYPD3 predicts a shallow minimum around the V···O_THF_ distance of 2.6 Å. However, upon elongation of the V···O_THF_ distance, the THF begins to rotate from “vertical” to “horizontal” orientation and reaches a more stable minimum along the V···O_THF_ direction at a V···O_THF_ distance of about 5 Å (structure B in Figure 5); upon releasing the O=V···O_THF_ constraint, the THF molecule displaces “horizontally” as to further optimize the F···H interactions (structure C in Figure 5). It is worth noting that CCSD also predicts that the standalone “horizonal” structure is more stable than the “vertical” one (Table 1).

**Figure 6:**
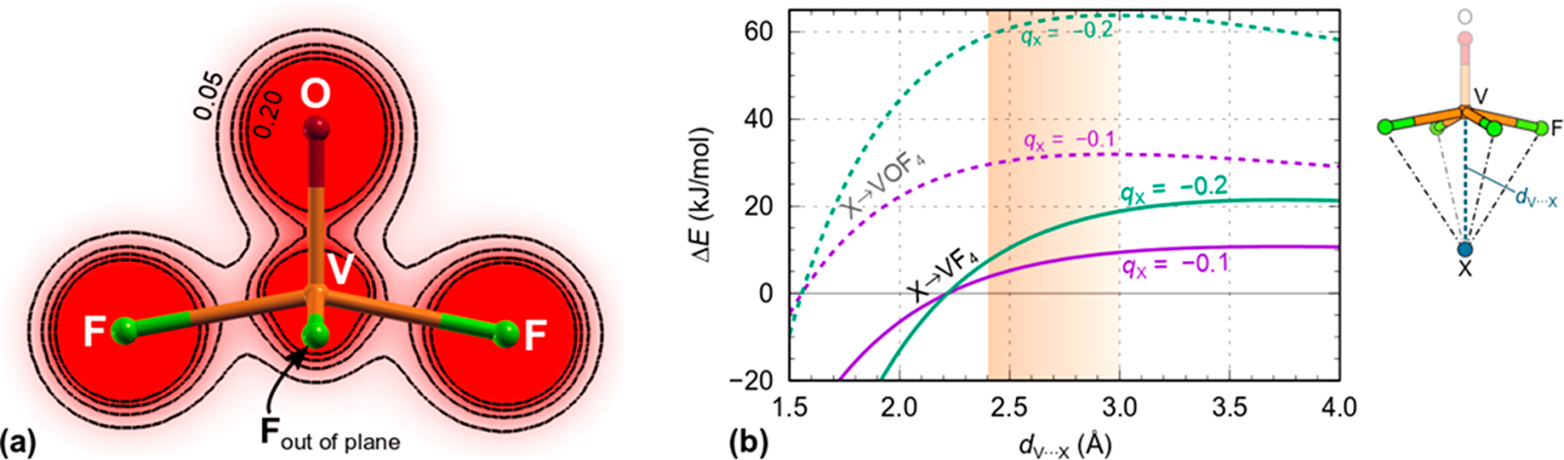
(a) Valence charge density of isolated VOF_4_^–^ plotted on the F–V(O)–F plane. Contours are drawn on the linear scale from 0 to 0.2 e/Bohr^3^ with an increment of 0.05 e/Bohr^3^. Notice a high valence electron density around the V nucleus, although V is formally in a +5 oxidation state; the corresponding Bader charges are +2.318 for V, −0.644 for F, and −0.743 for O. (b) Solid curves labeled X→VF_4_ represent the Coulomb interaction between the equatorial VF_4_ fragment of VOF_4_^–^ (modeled as point charges with aforementioned Bader values) and the negatively charged atom of a molecule (labeled as X and modeled by a fractional negative point charge of −0.1 and −0.2); if also the negatively charged O atom of the VOF_4_^–^ anion is taken into account, then obviously the interaction is more repulsive (dashed curves labeled X→VOF_4_). The orange band indicates the range of V···X distances that appear in crystal or standalone structures of [VOF_4_(L)]^−^ (L = THF or Py).

**Figure 7:**
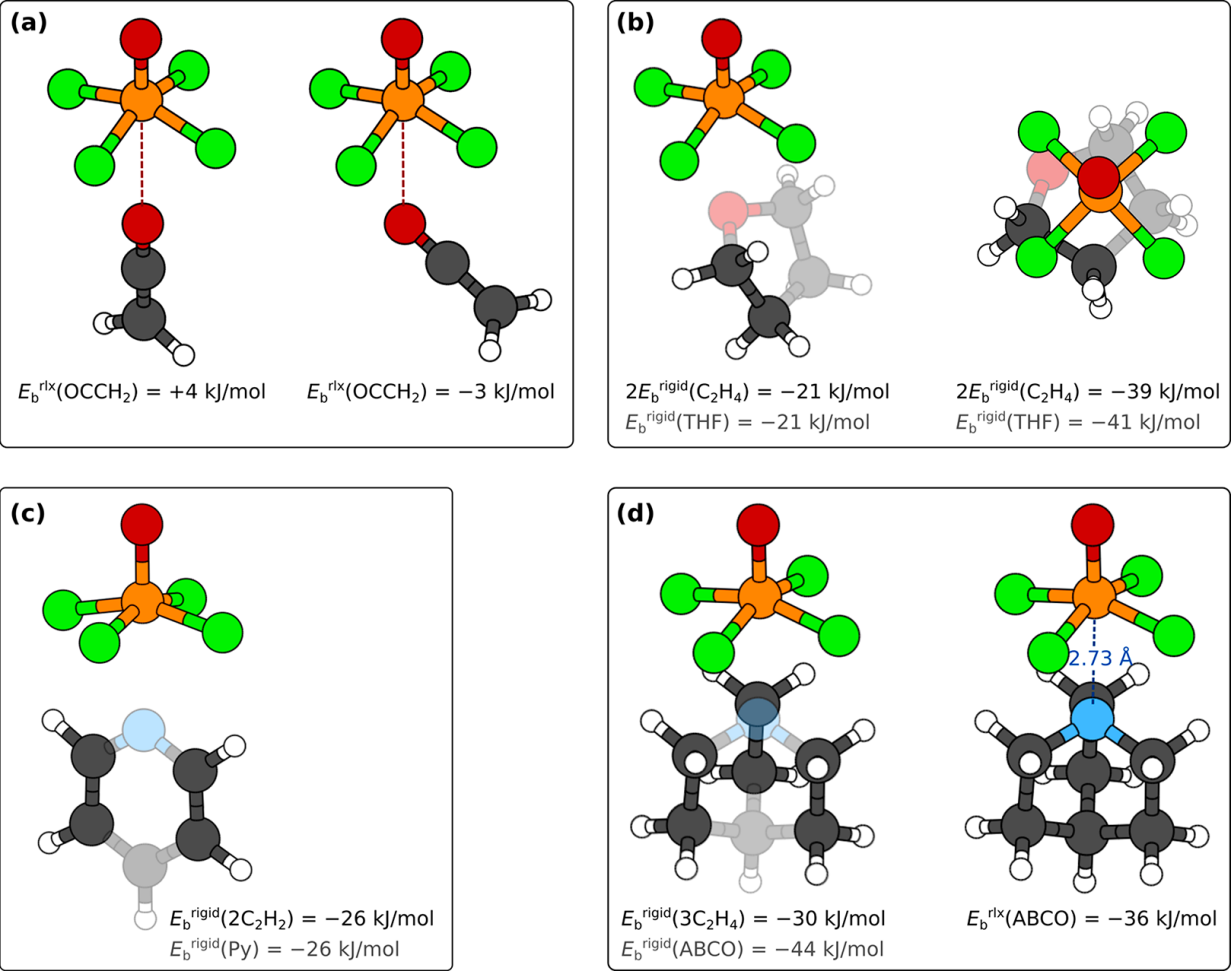
(a) Estimation of the V···O bonding with the ethenone molecule (H_2_C=C=O), whose structure is such that the F···H distances are very long (structures with the O=V···O_ethenone_ angles constrained to 180° and 120° are shown); *E*_b_^rlx^ values are estimated analogously to eq 1. (b–d) Estimation of the F···H interactions with distorted hydrocarbon fragments (drawn as emphasized) having a geometry compatible to that of a ligand (shaded) in the (b) [VOF_4_(THF)]^−^, (c) [VOF_4_(Py)]^−^, and (d) [VOF_4_(ABCO)]^−^ complexes. *E*_b_^rigid^ values are estimated analogously to eq 4. In d, also the structure of the relaxed [VOF_4_(ABCO)]^−^ complex along with the PBE-D3 calculated N_ABCO_–V distance and the relaxed binding energy *E*_b_^rlx^, eq 1, are reported. For the [VOF_4_(THF)]^−^ and [VOF_4_(ABCO)]^−^ complexes, the F···H interactions were estimated with distorted ethylene fragments (C_2_H_4_), whereas distorted acetylene fragments (C_2_H_2_) were used for the [VOF_4_(Py)]^−^ complex. All reported binding energies were calculated at the PBE-D3/def2TZVP level of theory.

Our analysis suggests that the interaction between the THF and VOF_4_^–^ is mainly driven by the electrostatic attraction between the negatively charged F atoms of VOF_4_^–^ and the positively charged H atoms of THF, because the weak bonding between the V(+5) center and negatively charged O_THF_, evidenced by the electron density difference plot in Figure 4, largely cancels with the electrostatic repulsion between the negatively charged O_THF_ atom and the negatively charged F atoms (*vide infra*). In order to explain this electrostatic repulsion between O_THF_ and VOF_4_^–^, we first need to recognize that the charge of the positively charged V atom is significantly smaller than its formal oxidation charge of +5. This is demonstrated by Figure 6a that shows a high valence electron density around the V nucleus. Calculated Bader charges of VOF_4_^–^ are +2.318 for V, −0.644 for F, and −0.743 for O.^27^ This implies that the positive charge of the V cation does not compensate for the negative charge of four equatorial F atoms (4 × (−0.644) = −2.576), and as a consequence the Coulomb interaction between negatively charged O_THF_ and VOF_4_^–^ is repulsive (Figure 6b). As a further crude estimate of the total binding of O_THF_ with VOF_4_^–^, we calculated the O···VOF_4_^–^ interaction with ethenone (H_2_C=C=O, Figure 7a), because its structure is such that the resulting F···H distances are very long (i.e., 6.3 Å for linear H_2_C=C=O···VOF_4_^–^ geometry). The PBE-D3 calculated interaction energy for linear H_2_C=C=O···VOF_4_^–^ geometry is marginal, +4 kJ/mol. However, the O atom of ethenone is sp^2^ hybridized, hence the tilted H_2_C=C=O···VOF_4_^–^ geometry seems more reasonable. The PBE-D3 calculation, with the O=V···O_ethenone_ angle fixed to 120° (the angle has to be constrained, otherwise the ethenone flips around so that the CH_2_ group points toward the negatively charged F atoms), gives a binding energy of −3 kJ/mol, but for this titled structure the F···H distances are considerably shorter (4.5 Å) compared to those in the linear structure. Both binding energies are thus close to zero and therefore corroborate the inference that the two involved interactions, i.e., the weak V···O_THF_ bond seen in the electron density difference plot (Figure 4) and the electrostatic repulsion between O_THF_ and the negatively charged F atoms (Figure 6b), cancel each other out. Furthermore, in the next two paragraphs, we will present two further arguments that the overall binding between VOF_4_^–^ and THF is indeed well accounted for only by attractive F···H interactions.

**Figure 8:**
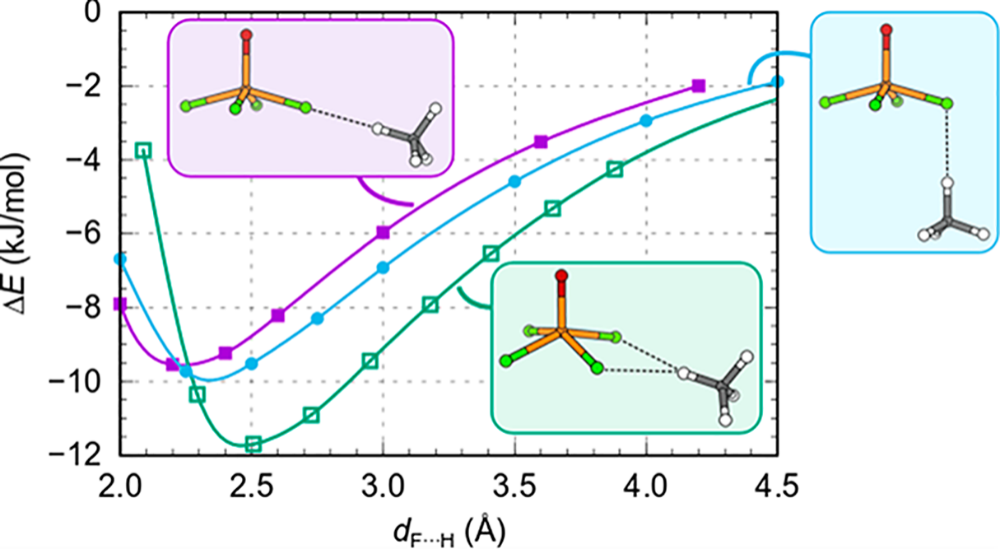
PBE-D3/def2TZVP calculated interaction energies between VOF_4_^–^ and methane (CH_4_) in various geometries as a function of the F···H distance. These calculations are used to estimate the strength of the linear and bifurcated F···H–C bonds.

To estimate the electrostatic attraction between the negatively charged F atoms of VOF_4_^–^ and the positively charged H atoms of THF, we performed a series of calculations between VOF_4_^–^ and methane (CH_4_) in various geometries as to model linear and bifurcated F···H–C bonds. Figure 8 reveals that F···H–C interaction can reach up to about −10 kJ/mol (−12 kJ/mol) for linear (bifurcated) bonds. The F···H–C interactions are long ranged; e.g., at an F···H distance of 4 Å there is still about −3 kJ/mol of interaction. The F···H–C interactions can therefore account for the binding between VOF_4_^–^ and THF for both “vertical” and “horizontal” geometry and also explain why the latter is more stable. That is, for “horizontal” geometry there are four bifurcated F···H–C bonds about 2.7 Å long, and by taking from Figure 8 the value of the bifurcated hydrogen bond strength at 2.7 Å (−11 kJ/mol), we can estimate the interaction to 4 × (−11) kJ/mol = −44 kJ/mol, which is similar to the PBE-D3 calculated binding energy of −39 kJ/mol between VOF_4_^–^ and THF in the “horizontal” structure (cf. Table 1). In the “vertical” geometry, there are two bifurcated F···H–C interactions and also two significantly longer linear F···H–C “bonds” (Figure 5, structure A). Utilizing the data from Figure 8 for the bifurcated and linear hydrogen bonds at respective bond distances of the “vertical” structure gives a value of about −35 kJ/mol, which is even significantly stronger than the PBE-D3 calculated interaction (cf. Table 1).

The second utilized approach to estimate the electrostatic attraction between the negatively charged F atoms of VOF_4_^–^ and the positively charged H atoms of THF was to model this interaction with hydrocarbon fragments having the same geometry as the [VOF_4_(THF)]^−^ complex. The concept along with the resulting interaction energies are schematically shown in Figure 7b. In particular, the F···H interactions were estimated by distorted ethylene fragments (C_2_H_4_). The resulting interaction energies are −21 and −39 kJ/mol for the “vertical” and “horizontal” geometries, respectively. These estimations are therefore similar to the aforementioned values obtained from the calculations between VOF_4_^–^ and CH_4_ and corroborate the assertion that the binding between THF and VOF_4_^–^ is mainly given by the attractive interaction between negatively charged F atoms and positively charged H atoms, whereas the weak covalent-like bonding between O_THF_ and V, seen in the electron density difference plot of Figure 4, cancels out with the electrostatic repulsion between O_THF_ and VOF_4_^–^. That the interaction between the ligand’s O heteroatom and the VOF_4_^–^ anion is indeed close to zero was shown above by means of the ethenone ligand (cf. Figure 7a).

The above analysis therefore explains why for the standalone [VOF_4_(THF)]^−^ complex the “horizontal” structure is more stable than the “vertical” structure. Namely, the binding between THF and VOF_4_^–^ is mainly given by the attractive F···H interactions, and the geometry of the “horizontal” structure is such that it optimizes the F···H interactions. This argument also explains why the geometry of standalone [VOF_4_(Py)]^−^ is vertical. Namely, Py is a planar molecule, and the F···H interactions are optimal in the predicted “vertical” geometry, because in this geometry two positively charged H atoms point toward the negatively charged F atoms of VOF_4_^–^, whereas in the “horizontal” geometry the H atoms would point away from the F atoms. The estimation of the F···H interactions with the aid of distorted-acetylene fragments yields a value of −26 kJ/mol for the “vertical” structure (Figure 7c), which implies that the binding between the Py ligand and the VOF_4_^–^ anion is almost exclusively given by the attractive interaction between negatively charged F atoms and positively charged H atoms, even though the electron density difference plot (Figure 4) suggests that the V···N_Py_ interaction of [VOF_4_(Py)]^−^ is stronger than the V···O_THF_ interaction of [VOF_4_(THF)]^−^. This is consistent with the observation that N_Py_ is more negatively charged than O_THF_. The respective Bader charges are −2.66 and −1.52, hence the electrostatic repulsion between the electronegative heteroatom of the ligand and the F atoms of the anion is larger for Py.

To further corroborate the claim that the interaction between a ligand and the VOF_4_^–^ anion is mainly driven by the electrostatic F···H interactions, we now consider an even more nucleophilic ligand than Py, in particular, 1-azabicyclo[2.2.2]octane (ABCO). Calculations indicate that ABCO is chemically softer and less electronegative than Py (Table S7), thus suggesting that it is a stronger Lewis base than Py. According to calculations, ABCO binds stronger to the VOF_4_^–^ anion than Py. The PBE-D3 calculated binding energy is −35 kJ/mol for the standalone [VOF_4_(ABCO)]^−^ complex (Table 1), whereas the estimation of the F···H interactions with aid of distorted ethylenes yields a value of −30 kJ/mol (Figure 7d). This implies that, even in this case, the binding of the ligand to the VOF_4_^–^ anion mainly stems from the electrostatic F···H interactions.

**Figure 9:**
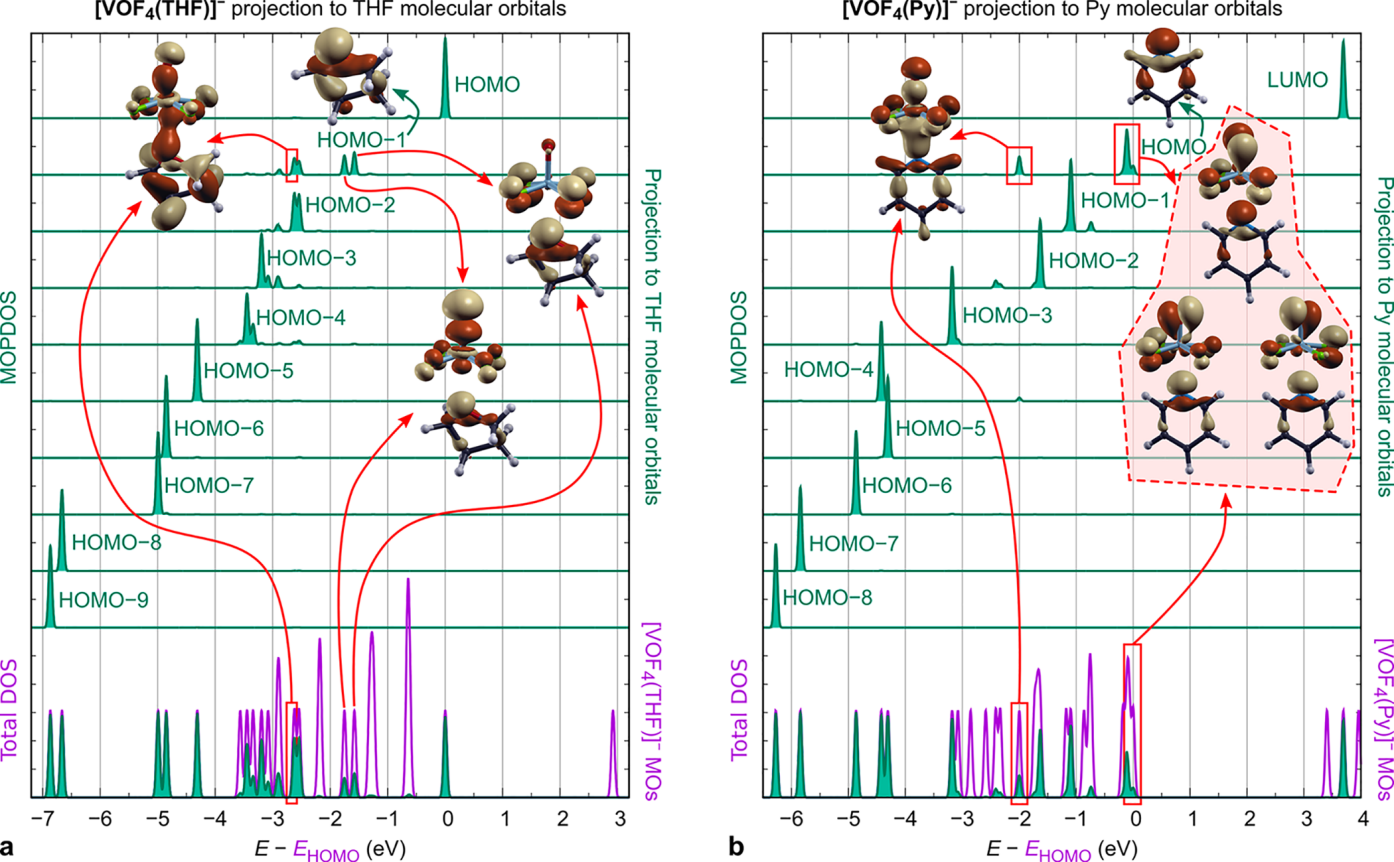
Density of states (DOS) analyses of the standalone (a) [VOF_4_(THF)]^−^ and (b) [VOF_4_(Py)]^−^ complexes. The purple curves at the bottom of the plots represent the total DOS, whereas the superposed green curves are the DOS projections to the (a) THF and (b) Py fragments. Above these curves, the DOS is projected to the individual molecular orbitals of (a) THF and (b) Py fragments (the projections to the VOF_4_^–^ anion are shown in Figure S1). For THF, its HOMO–1 orbital is involved in the interaction with the VOF_4_^–^ anion, whereas for Py the corresponding orbital is HOMO. Both ligands form one bonding state with the VOF_4_^–^ anion and either two (THF) or three (Py) states that are more antibonding in character. The snapshots of the corresponding signed molecular orbital densities, sgn(ψ_*i*_(**r**))|ψ_*i*_(**r**)|^2^, as well as those corresponding to the HOMO–1 of THF and HOMO of Py are also shown. The label HOMO–*n* stands for the *n*th orbital below HOMO.

**Figure 10:**
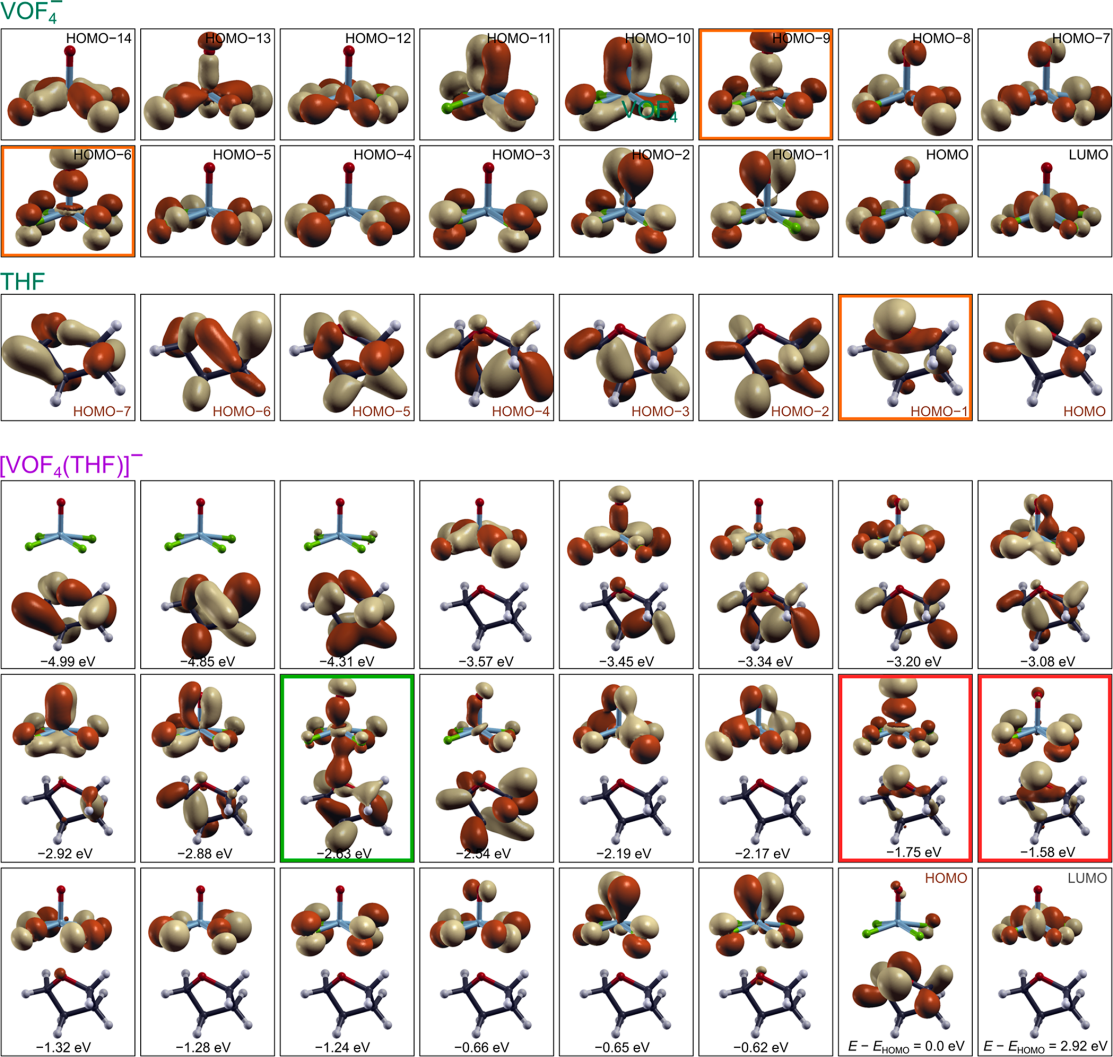
A subset of PBE-D3 calculated signed molecular orbital densities, sgn(ψ_*i*_(**r**))|ψ_*i*_(**r**)|^2^, of the VOF_4_^–^ anion, the THF ligand, and the standalone [VOF_4_(THF)]^−^ complex. There is only one bonding molecular orbital between VOF_4_^–^ and THF, which is highlighted with a green rectangle, whereas antibonding molecular orbitals between the two fragments are highlighted by red rectangles. Molecular orbitals of the individual VOF_4_^–^ and THF fragments that are predominantly involved in the highlighted states are marked with orange rectangles. Eigenvalues of the molecular orbitals, measured with respect to the HOMO eigenvalue (*E*_HOMO_), are also given.

The lack of an effective chemical interaction between the heteroatom of a ligand and the VOF_4_^–^ anion is also qualitatively confirmed by the molecular orbital (MO) analysis. To help disentangle which MOs of a ligand and the VOF_4_^–^ anion are involved in the ligand–anion interaction, we utilized DOS projected to individual molecular orbitals of the involved fragments (MOPDOS). The corresponding projections to the ligands are shown in Figure 9 for the [VOF_4_(THF)]^−^ and [VOF_4_(Py)]^−^ complexes, whereas projections to the VOF_4_^–^ anion are shown in Figure S1 in the Supporting Information. For both cases, the MOPDOS analysis reveals no ligand-to-anion electron charge donation nor any anion-to-ligand backdonation. This is consistent not only with the results of Bader analysis, according to which the charges of THF and Py ligands in the two complexes are very close to zero, i.e., +0.007 and +0.036, respectively, but also with V(+5) being a hard Lewis acid. As for the [VOF_4_(THF)]^−^ complex (Figure 9a), the interaction between THF and VOF_4_^–^ involves one bonding state between the two fragments, located 2.6 eV below the HOMO eigenvalue (*E*_HOMO_), that is predominantly due to mixing of the HOMO–1 orbital of THF (Figure 9a) with the HOMO–9 orbital of VOF_4_^–^ (Figure S1a). However, this bonding state is counteracted by two antibonding states, located 1.8 and 1.6 eV below *E*_HOMO_, that mainly stem from mixing of the HOMO–1 orbital of THF (Figure 9a) with the HOMO–6 and HOMO–9 orbitals of VOF_4_^–^ (Figure S1a). The snapshots of the signed molecular orbital densities corresponding to these three states are also shown in Figure 9a, whereas Figure 10 plots a much larger subset of valence signed molecular orbital densities of the VOF_4_^–^ and THF fragments as well as of the [VOF_4_(THF)]^−^ complex (the corresponding plots for the [VOF4(Py)]^−^ complex are shown in Figure S2 in the Supporting Information).

Also for the [VOF_4_(Py)]^−^ complex (Figure 9b), the interaction between the ligand and the anion involves the bonding and antibonding states that are all occupied. In particular, the Py–VOF_4_^–^ interaction involves one bonding state, located 2.0 eV below *E*_HOMO_, that is due to mixing of the HOMO orbital of Py (Figure 9b) with the HOMO–9 orbital of VOF_4_^–^ (Figure S1b). However, this bonding state is counteracted by the three highest occupied states that display antibonding character and stem from the interaction of the Py HOMO orbital (Figure 9b) with the HOMO–9, HOMO–2, HOMO–1, and HOMO orbitals of VOF_4_^–^ (Figure S1b). The snapshots of the signed molecular orbital densities corresponding to these four states are also shown in Figure 9b (see also Figure S2 for a much larger subset of valence signed molecular orbital densities). The fact that both bonding and antibonding molecular orbitals, relevant for the ligand–anion interaction, are occupied is consistent with the lack of effective chemical interaction between the heteroatom of a ligand and the metal center and further corroborates our claim that the ligand–anion binding is by and large due to the attractive electrostatic F···H interactions.

#### 3.2.4 Chemistry of the F···H–C Bonding

We should comment on the nature of the F···H–C interactions in the [VOF_4_(THF)]^−^ and [VOF_4_(Py)]^−^ complexes. The analysis of the electron density difference (cf. Figure 4) reveals that there is neither any electron accumulation in between the F···H contacts nor any electron polarization in the F···H direction (even at contours/isovalues much lower than those shown in Figure 4, we observe no such electron accumulation or polarization). Note from Figure 4 that small electron accumulation lobes at negatively charged F atoms are instead polarized toward the O_THF_ or N_Py_ atoms. Figure 4 therefore suggests that the F···H interaction is purely electrostatic without any covalent contribution. This is consistent with the knowledge that fluorine is not a good H-bond acceptor,^60,61^ although metal fluorides can form strong hydrogen bonds,^62^ but not with the F···H–C contacts.

#### 3.2.5 Other Solvent Molecules

**Figure 11:**
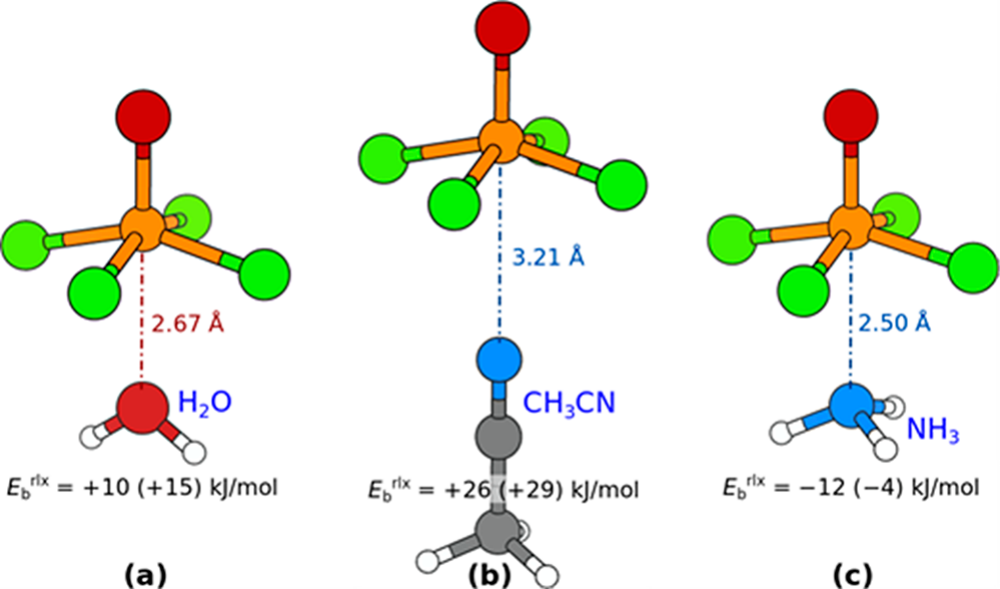
None of the presented standalone (a) [VOF_4_(H_2_O)]^−^, (b) [VOF_4_(CH_3_CN)]^−^, and (c) [VOF_4_(NH_3_)]^−^ complexes in “vertical” geometry oriented with the O or N atom toward the V atom is stable. Presented structures were obtained by constraining the O=V···X (X = O or N) angle to 180° (and not allowing the H atoms to flip around for H_2_O molecule)—without the imposed constraint the molecules would flip around. PBE-D3/def2TZVP calculated binding energies, calculated analogously to eq 1, are also given (BSSE corrected values are stated in parentheses). The corresponding fully relaxed structures (without any constraint) are shown in Figure 12.

**Figure 12:**
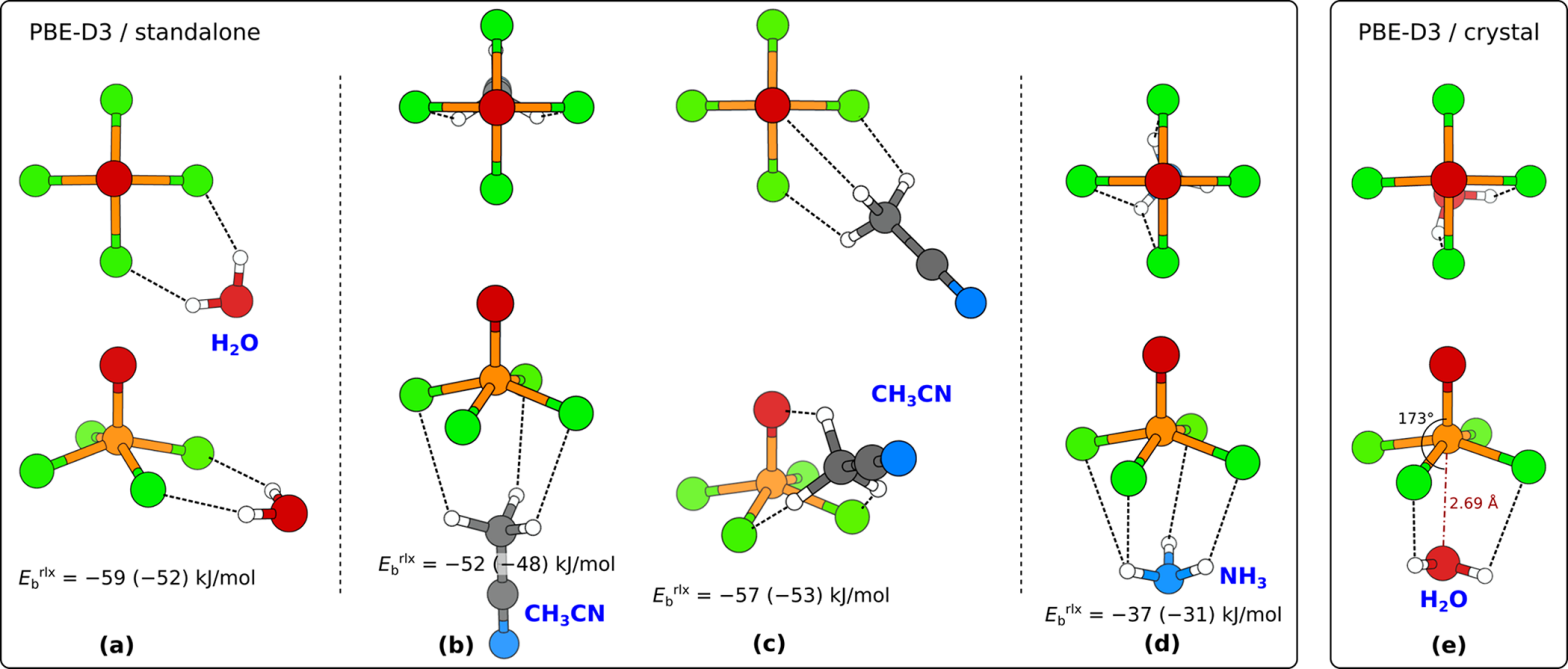
Top-view (top row) and perspective-view (bottom row) snapshots of various optimized [VOF_4_(L)]^−^ complexes. Structures of standalone complexes, calculated with the PBE-D3/def2TZVP method: (a) [VOF_4_(H_2_O)]^−^, (b,c) [VOF_4_(CH_3_CN)]^−^, and (d) [VOF_4_(NH_3_)]^−^. Binding energies, calculated analogously to eq 1, are also given (BSSE corrected values are stated in parentheses). (e) Structure of the [VOF_4_(H_2_O)]^−^ complex within the crystal, calculated with PBE-D3/plane-wave method (for clarity, atoms surrounding the complex are not shown). Rigid binding energies of H_2_O within the crystal are provided in Table 2.

We also calculated the ligand–VOF_4_^–^ interaction with some other typical solvent molecules that often act as ligands, in particular, water (H_2_O), acetonitrile (CH_3_CN), and ammonia (NH_3_), to see whether these can bind with the O or N atom to the V cation. According to the above argument that the interaction between the molecule and VOF_4_^–^ anion is almost exclusively driven by F···H interactions, they should not. Instead, these molecules should flip around as to interact with H atoms. This is precisely what the calculations predict, because all the “vertical” [VOF_4_(L)]^−^ complexes oriented with O or N atom toward the V cation are found unstable (Figure 11). Be aware that the “vertical” structures and binding energies shown in Figure 11 were obtained by constraining the O=V···X (X = O or N) angle to 180° and not allowing the H atoms to flip around for the H_2_O molecule. Notice that among the shown “vertical” structures only the binding energy for ammonia complex is slightly exothermic, whereas the values for water and acetonitrile are endothermic (the reason that the molecules are trapped at about 2.8 ± 0.4 Å from the V cation is that at this distance there is a shallow minimum in the constrained O=V···X direction). When the constraint is removed, the molecules flip around during the relaxation (H_2_O and CH_3_CN also reposition) as to optimize the F···H interactions (Figure 12). In the standalone complexes, H_2_O shifts from the sixth octahedral position toward the equatorial plane of F atoms and forms two linear F···H bonds with two negatively charged F atoms with a net binding energy of about −50 kJ/mol (Figure 12a). Acetonitrile displays two local minima, and for both of them it is oriented with methyl toward the VOF_4_^–^ anion. In the first, slightly less stable, local minimum, it remains in the sixth octahedral position, but with methyl oriented toward the equatorial plane of negatively charged F atoms (Figure 12b), whereas in the second, more stable, minimum, the CH_3_CN ligand is located above the plane of equatorial F atoms such that it faces with methyl toward the octahedral plane spanned by O and two negatively charged F atoms (Figure 12c). The binding energy for both modes is about −50 kJ/mol. Ammonia displays analogous local minima as CH_3_CN, but for NH_3_ the latter minimum is slightly less stable (not shown) compared to the first one (Figure 12d). Ammonia is well-known to display no propensity to act as a hydrogen bond donor,^63^ yet despite this fact it still prefers to interact with the VOF_4_^–^ anion via F···H interactions rather than the V···N_NH3_ bonding (the binding energy for the latter mode is only about −10 kJ/mol, Figure 11c).

Finally, we also tested if the crystal packing effects can stabilize the “vertical” H_2_O···VOF_4_^–^ geometry with O_H_2_O_ oriented toward the V center and H atoms away from it (this geometry is shown in Figure 11a). To this end, we utilized the optimized crystal structure of [(L^Dipp^)H][VOF_4_(THF)] (**1**) and replaced all the THF molecules with H_2_O molecules such that all [VOF_4_(H_2_O)]^−^ complexes displayed the “vertical” structure of Figure 11a. After the variable-cell optimization (the corresponding lattice parameters are tabulated in Table S6), the O=V···O_H_2_O_ angle remained close to 180°; however, the H_2_O molecules rotated by about 90° such that H atoms up-shifted as to interact with two negatively charged F atoms (Figure 12e), but the O_H_2_O_ atom remained at a similar position, located 2.69 Å from the V cation. This is a rather notable result, because experimentally it is difficult to detect H atoms. And, if such a position of O_H_2_O_ near a multivalent cation would be detected, one would be tempted to draw the H_2_O ligand in a tilted “tetrahedral” geometry with a V···O–H angle of about 110° (e.g., see Figure 12 of ref (32)), hence with the H atoms pointing away from the metal center, which is currently not the case.

The PBE-D3/plane-wave calculated rigid binding energy of H_2_O within the hypothetical [(L^Dipp^)H][VOF_4_(H_2_O)] crystal structure is −42 kJ/mol (Table 2), out of which two-thirds (−28 kJ/mol) are due to direct H_2_O–VOF_4_^–^ interaction. Hence, for H_2_O, the crystal packing effects contribute less to the overall binding than for THF and Py (Table 2), which can be attributed to a smaller size of H_2_O compared to THF and Py (i.e., London dispersion interactions are weaker for smaller molecules). Nevertheless, crystal packing effects are still sufficiently “strong” to hold H_2_O at the sixth octahedral position, because in the standalone [VOF_4_(H_2_O)]^−^ complex, the water molecule displaces toward the plane of equatorial F atoms and forms two linear F···H bonds as shown by Figure 12a.

Although the finding that in all considered cases a ligand and anion are held together by F···H–C interactions (and by crystal-packing effects) and not by the heteroatom–metal center interaction may seem counterintuitive, it can be rationalized with the aid of the HSAB (hard and soft acids and bases) principle^64−66^ (some calculated electronic parameters of the considered ligands, relevant for HSAB, are reported in Table S7 in the Supporting Information). Namely, the V(+5) metal center is a hard Lewis acid, and hard acids prefer electrostatic interactions.^65,66^ This suggests that the interaction between a ligand and VOF_4_^–^ should be dominated by electrostatics. While the ligands’ O and N heteroatoms are negatively charged and therefore of appropriate sign to interact attractively with the V(+5) metal center, it is the geometry and the electronic structure of the VOF_4_^–^ anion that prevent this, because we showed that the heteroatom–V(+5) attraction is usually more than counteracted by the repulsion between the heteroatom and electronegative F atoms (Figure 6b). It is this cancellation between the heteroatom–V(+5) attraction and heteroatom–F repulsion that makes the electrostatic F···H–C attraction the dominant force determining the interaction between a ligand and VOF_4_^–^ in standalone complexes. This is why for most considered standalone complexes a ligand rotates with its heteroatom away from the metal center as to optimize the F···H–C contacts.

### 3.3 Other Oxohalido Anions

#### 3.3.1 Oxohalido Niobate Anions

**Figure 13:**
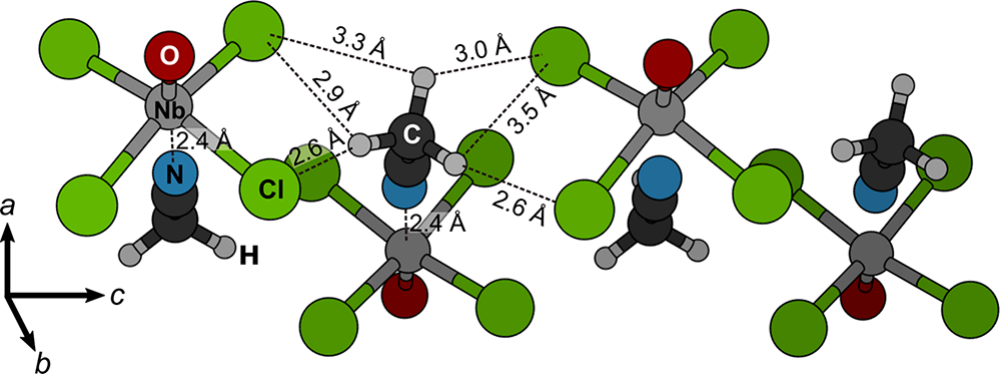
Alignment of CH_3_CN molecules and NbOCl_4_^–^ anions along the *c* axis in the crystal structure of [L^PPh3Me^][NbOCl_4_(CH_3_CN)]. CH_3_CN faces with the N_CH_3_CN_ atom toward the Nb metal center and with the methyl group toward the equatorial F atoms of the other two neighboring NbOCl_4_^–^ anions, such that each H atom forms a bifurcated Cl···H–C bond. PBE-D3/plane-wave calculated F···H and N–Nb distances are also given.

Crystal structures of closely related [NbOCl_4_]^−^ and [NbOBr_4_]^−^ anions with THF, Py, CH_3_CN, and H_2_O molecules located at the sixth octahedral position and oriented with their heteroatom toward the niobium metal center exist in the literature.^18,20,23−25^ Could the above hypothesis that the molecule is held in place due to crystal packing effects and electrostatic X···H–C interactions explain also the bonding in these compounds? To shed some light on this question, we analyzed the bonding in the crystal structure of [L^PPh3Me^][NbOCl_4_(CH_3_CN)]^18,20,23−25^ (L^PPh3Me^ = methyltriphenylphosphonium). The PBE-D3/plane-wave calculated crystal structure of [L^PPh3Me^][NbOCl_4_(CH_3_CN)] is in very good agreement with the experimental one (Table S6 in the Supporting Information). In this compound, CH_3_CN is oriented with its N_CH_3_CN_ heteroatom toward the Nb metal center, but in line with the aforementioned arguments, also for this compound the PBE-D3 and B2PLYPD3 calculations indicate that the interaction between the CH_3_CN molecule and the NbOCl_4_^–^ anion is close to vanishing in the standalone [NbOCl_4_(CH_3_CN)]^−^ complex (Table 1). In contrast, the PBE-D3 calculated rigid binding energy of CH_3_CN in the crystal structure is a sizable −111 kJ/mol (Table 2). This value indeed stems mainly from the crystal packing effects and the Cl···H–C interactions. In the crystal structure, CH_3_CN is oriented with the N_CH_3_CN_ atom toward the Nb metal center and with the methyl group toward the equatorial Cl atoms of the other two neighboring NbOCl_4_^–^ anions (Figure 13). In addition, CH_3_CN molecules are aligned antiparallel and form a zigzag-like chain of dipoles along the *c* crystal axis (Figure 13); note that CH_3_CN has a large dipole moment (calculated value is about 4 D). Bonding analysis reveals that Cl···H–C interactions contribute about −20 kJ/mol and dipole–dipole interactions of the CH_3_CN zigzag chain about −10 kJ/mol, whereas the rigid binding energy of CH_3_CN–NbOCl_4_^–^ is about −15 kJ/mol (Table 2). The remaining −65 kJ/mol comes from other crystal packing effects, particularly from long-range electrostatic charge–dipole interactions (note that crystal consists of [L^PPh3Me^]^+^ cations, NbOCl_4_^–^ anions, and highly polar CH_3_CN).

#### 3.3.2 Other Oxohalido Vanadate Anions?

In contrast to [NbOCl_4_]^−^ and [NbOBr_4_]^−^, no such vanadium oxohalido anion crystal structure containing other halides than fluoride was found in the literature. Is the reason due to a reduced strength of X···H–C interactions (X = Cl, Br) compared to F···H–C ones? To answer this question, we calculated the strength of interaction between VOX_4_^–^ (X = Cl, Br, and I) and methane (CH_4_) in various geometries as a function of the X···H distance, analogously to what was done for F···H in Figure 8, and the results are shown in Figure S3 in the Supporting Information. These calculations were used to estimate the strength of the linear and bifurcated X···H–C contacts. Results show that such interactions do exist in all cases, but they are weaker than F···H, in particular Cl···H is about 20%, Br···H is about 30%, and I···H is about 40% weaker than F···H. The reduced strengths may therefore indeed contribute to the lack of such crystal structures in the literature. The other reason can be attributed to smaller affinity of the V metal center (compared to Nb) toward the neutral ligands—note that V(+5) is a harder Lewis acid than Nb(+5) (see Tables S8 and S9)—as deduced from the bonding analysis of crystal structures of [L^PPh3Me^][NbOCl_4_(CH_3_CN)] and hypothetical [L^PPh3Me^][VOCl_4_(CH_3_CN)] (Table 2) as well as the corresponding [NbOCl_4_(CH_3_CN)]^−^ and [VOCl_4_(CH_3_CN)]^−^ standalone complexes (Table 1); the hypothetical crystal structure of the V analogue was calculated by replacing Nb with V in the crystal structure of [L^PPh3Me^][NbOCl_4_(CH_3_CN)] and performing a variable-cell relaxation (the resulting crystal structure data are given in Table S6).

## 4 Conclusions

Crystal structure analysis is currently the most important characterization method in synthetic chemistry. Solved crystal structure models help chemists explain reactions, mechanisms, and interactions. However, relying solely on crystal structures can lead toward the wrong interpretation of bonding. We showed that newly determined crystal structures of [VOF_4_(L)]^−^ (L = THF, Py) clearly reveal the “coordination” of the neutral ligand to the metal center of the anion due to their mutual position and orientation of the ligand. Hence, it would be reasonable to conclude that there is a chemical bonding between the ligand and the metal center. Nonetheless, according to the quantum chemical calculations, such a conclusion is wrong. Notably, calculations of the standalone [VOF_4_(THF)]^−^ complex unexpectedly resulted in the ligand molecule turning with its heteroatom away from the vanadium center in order to increase the number of F···H–C interactions, and periodic-boundary calculations of the whole crystal structure were required to reproduce the experimentally determined structure in good agreement. This implies that in the crystal structure the ligand is held in place by the crystal packing effects, which, according to the bonding analysis, contribute about three-quarters to the overall bonding. The other contribution to bonding comes from the F···H–C interactions between the anion and the ligand molecule, which for isolated complexes become the dominant force that keeps the two together. In contrast, the interaction between the metal center and electron rich heteroatom of the ligand cancels out by the repulsion between the negatively charged oxygen and fluorine atoms of the anion and the heteroatom of the ligand. Further analyses of oxohalido vanadate and oxohalido niobate anions and their interactions with several other neutral ligands reveal similar ligand–anion bonding trends. The obstacle that restricts the abundance of such compounds with oxohalido MOX_4_ anions (for X = Cl, Br, or I) is the reduced strength of the X···H interactions that are weaker than F···H. In particular, Cl···H is about 20%, Br···H is about 30%, and I···H is about 40% weaker than F···H. The reduced strengths may therefore indeed contribute to the lack of such crystal structures in the literature. As for the lack of compounds with vanadate [VOCl_4_]^−^ or [VOBr_4_]^−^ anions, another reason is that the vanadium metal center displays smaller affinity than niobium toward neutral ligands.

The knowledge we have obtained through this research shows that, when elucidating the structure, it can be misleading to rely solely on common aspects of the coordination chemistry, such as ligand orientation and bond distances, because they may lead to incorrect conclusions and a diminished value of research.

## Supporting Information Available

Additional structural data and DFT calculation data (PDF)

## Accession Codes

CCDC 2065279–2065280 contain the supplementary crystallographic data for this paper. These data can be obtained free of charge via www.ccdc.cam.ac.uk/data_request/cif, or by emailing data_request@ccdc.cam.ac.uk, or by contacting The Cambridge Crystallographic Data Centre, 12 Union Road, Cambridge CB2 1EZ, UK; fax: +44 1223 336033.

## Abbreviations

THF: tetrahydrofuran
Py: pyridine
ABCO: 1-azabicyclo[2.2.2]octane
DOS: density of states
MOPDOS: molecular-orbital projected DOS
