# Supplementary material for: Coordination of a Neutral Ligand to a Metal Center of Oxohalido Anions: Fact or Fiction?
Source: Inorg Chem. 2021 Aug 5;60(16):11932–47. doi: 10.1021/acs.inorgchem.1c00947 (PMC8388118; doi:10.1021/acs.inorgchem.1c00947)
Supplement: Supplementary file 1 — ic1c00947_si_001.pdf [file ic1c00947_si_001.pdf]

Coordination of a neutral ligand to a metal center of oxohalido anions: fact or fiction?

Supporting information

Anton Kokalj,[a,c] Žiga Zupanek,[b,c] Melita Tramšek[b] and Gašper Tavčar[b,c*]

[a] Department of Physical and Organic Chemistry, Jožef Stefan Institute, Jamova 39, 1000 Ljubljana, Slovenia

[b] Department of Inorganic Chemistry and Technology, Jožef Stefan Institute, Jamova 39, 1000 Ljubljana, Slovenia

[c] Jožef Stefan International Postgraduate School, Jamova 39, 1000 Ljubljana, Slovenia

Table of content

S1. Crystal structure analysis 2
S2. Comparison of experimental and DFT calculation data 7
S3. MOPDOS of the $[VOF_4(THF)]^-$ and $[VOF_4(Py)]^-$ complexes: projections to the VOF_4^- anion 8
S4. Molecular orbitals of the VOF_4^- anion, Py ligand, and $[VOF_4(Py)]^-$ complex 9
S5. Some electronic parameters of ligands and metal centers 10
S6. Estimation of the X···H−C bond strengths 12
S7. References 13

S1. Crystal structure analysis

Table S1. Summary of crystal data and structure refinement for $[(L^{Dipp})H][VOF_4(THF)]$ (**1**) and $[(L^{Dipp})H][VOF_4(Py)]$ (**2**).

Compound	$[(L^{Dipp})H][VOF_4(THF)]$ **(1)**	$[(L^{Dipp})H][VOF_4(Py)]$ **(2)**
CCDC No.	2065280	2065279
Formula	$C_{27}H_{37}N_2{\cdot}C_4H_8F_4O_2V$	$2(C_{27}H_{37}N_2){\cdot}2(C_5H_5F_5NOV)$
F_w	604.63	1223.25
T [K]	150	150
Crystal system	Monoclinic	Triclinic
Space group	$P2_1/c$	$P-1$
a [Å]	12.6595(1)	12.5745(3)
b [Å]	12.7750(1)	12.7175(3)
c [Å]	19.6070(2)	20.5561(5)
α [°]	90	88.902(2)
β [°]	93.396(1)	81.187(2)
γ [°]	90	87.142(2)
V [Å^3]	3165.38(5)	3244.2(1)
Z	2	2
ρ_{calc} [g/cm^3]	1.269	1.252
Crystal size [mm]	0.49×0.35×0.27	0.68×0.29×0.17
Radiation type	Cu Kα	Cu Kα
λ [Å]	1.54184	1.54184
μ [mm^{-1}]	3.066	2.986
F(000)	1280	1288
Θ_{max} [°]	67.080	67.073
	$-15 \leq h \leq 15$	$-14 \leq h \leq 15$
Index ranges	$-15 \leq k \leq 15$	$-15 \leq k \leq 15$
	$-23 \leq l \leq 23$	$-24 \leq l \leq 24$
Reflections collected	71450	34582
Independent reflections	5637	11479
Reflections with $[I > 2\sigma(I)]$	5262	10344
R_{int}	0.0330	0.0228
R_{sigma}	0.0117	0.0220
Data/restrains/parameters	5637/13/415	11479/0/755
S[a]	1.090	1.032
R_1[b], wR_2[c] $[I > 2\sigma(I)]$	0.0376, 0.1004	0.0398, 0.1087
R_1[b], wR_2[c] [all data]	0.0400, 0.1019	0.0449, 0.1131
$\Delta\rho_{min}$, $\Delta\rho_{max}$ [eÅ^{-3}]	−0.356, 0.412	−0.397, 0.684

[a] $S = [\Sigma(w(F_o^2-F_c^2)^2)/(N_o-N_p)]^{1/2}$.
[b] $R_1 = ||F_o|-|F_c||/\Sigma|F_o|$.
[c] $wR_2 = [\Sigma(w(F_o^2-F_c^2)^2)/\Sigma(w(F_o^2)^2)]^{1/2}$.

Table S2. Bond lengths [Å] and angles [°] for both domains of the [(L^{Dipp})H][VOF_4(THF)] (**1**) as well as the occupancy for domain A and B.

Domain of THF	Occupancy	
A	46.1(9) %	
B	53.9(9) %	
**Intramolecular bond**	**Bond length [Å]**	**Bond valence[a]**
V=O	1.561(2)	1.923
V−O_{THF}	2.403(7) (O30A; domain A)<br>2.464(6) (O30B; domain B)	0.198 (O30A)<br>0.168 (O30B)
V−F	1.819(1) (F1)<br>1.805(1) (F2)<br>1.806(1) (F3)<br>1.821(1) (F4)	0.745<br>0.774<br>0.771<br>0.741
	Σs_V	5.152 (domain A)<br>5.122 (domain B)
**Intermolecular bond**	**Bond length [Å]**	
F−C2	3.004(2) (F1)<br>2.986(2) (F4)	
F−C4	3.936(2) (F2)<br>2.946(2) (F3)	
F−C5	3.081(2) (F2)<br>3.065(2) (F3)	
**Intramolecular angle**	**Angle [°]**	
O=V−F	99.64(7) (F1)<br>100.21(9) (F2)<br>100.53(7) (F3)<br>98.69(8) (F4)	
O_{THF}−V−F	82.1(2) (F1, O30A, domain A)<br>88.3(2) (F2, O30A, domain A)<br>77.7(2) (F3, O30A, domain A)<br>72.9(2) (F4, O30A, domain A)<br>76.3(2) (F1, O30B, domain B)<br>77.1(2) (F2, O30B, domain B)<br>83.6(2) (F3, O30B, domain B)<br>83.9(2) (F4, O30B, domain B)	
O=V−O_{THF}	171.3(2) (O30A, domain A)<br>175.1(2) (O30B, domain B)	
F−V−F_{cis}	88.89(6) (F1, F2)<br>86.60(5) (F1, F4)<br>88.95(6) (F2, F3)<br>88.96(5) (F3, F4)	
F−V−F_{trans}	159.78(6) (F1, F3)<br>161.05(7) (F2, F4)	
**Intermolecular angle**	**Angle [°]**	
$Plane_{equatorial\text{-}F}$−$Plane_{imidazolium}$	87.0(5) (C2 toward anion)<br>23.9(6) (C4, C5 toward anion)	

[a] Bond valence analyses were done using the equation $\sum_j s_{ij} = \sum_j e^{\left(\frac{R_{ij}^0 - R_{ij}}{B}\right)}$, with parameters $R_{(V–O)}$ = 1.803 Å, $R_{(V–F)}$ = 1.71 Å, B = 0.37 Å.[1, 2]

Table S3. Bond lengths [Å] and angles [°] for both subunits of the $[(L^{Dipp})H][VOF_4(Py)]$ (**2**).

	Subunit 1		Subunit 2	
Intramolecular bond	Bond length [Å]	Bond valence[a]	Bond length [Å]	Bond valence[a]
V=O	1.570(2)	1.877	1.574(2)	1.857
$V-N_{Py}$	2.399(2)	0.233	2.406(2)	0.229
V−F	1.840(1) (F1)	0.704	1.832(1) (F5)	0.719
	1.804(1) (F2)	0.776	1.805(1) (F6)	0.774
	1.831(1) (F3)	0.721	1.821(1) (F7)	0.741
	1.811(1) (F4)	0.761	1.815(1) (F8)	0.753
	Σs_V	5.072	Σs_V	5.072
Intermolecular bond	Bond length [Å]		Bond length [Å]	
$F-C_{imidazolium}$	2.896(2) (F1, C2)		2.888(2) (F5, C31)	
	4.175(2) (F2, C2)		4.251(3) (F6, C31)	
	4.032(2) (F3, C33)		3.169(2) (F7, C4)	
	3.100(2) (F3, C34)		4.051(2) (F7, C5)	
	2.921(2) (F4, C33)		3.022(2) (F8, C4)	
	2.877(2) (F4, C34)		2.984(2) (F8, C5)	
Intramolecular angle	Angle [°]		Angle [°]	
O=V−F	99.45(9) (F1)		98.62(9) (F5)	
	99.49(8) (F2)		98.07(8) (F6)	
	99.13(9) (F3)		99.34(9) (F7)	
	98.61(9) (F4)		100.40(7) (F8)	
$N_{Py}-V-F$	80.70(6) (F1)		79.66(6) (F5)	
	80.14(6) (F2)		79.25(6) (F6)	
	80.73(6) (F3)		82.53(6) (F7)	
	81.75(6) (F4)		82.28(5) (F8)	
$O=V-N_{Py}$	179.61(9)		176.78(8)	
$F-V-F_{cis}$	89.34(6) (F1, F2)		89.54(7) (F5, F6)	
	86.75(5) (F1, F4)		87.53(6) (F5, F8)	
	89.00(6) (F2, F3)		90.52(7) (F6, F7)	
	89.08(5) (F3, F4)		86.69(6) (F7, F8)	
$F-V-F_{trans}$ [°]	161.37(6) (F1, F3)		161.85(7) (F5, F7)	
	161.87(7) (F2, F4)		161.53(6) (F6, F8)	
Intramolecular angle	Angle [°]		Angle [°]	
$Plane_{equatorial\ F}-Plane_{equatorial\ F}$	80.80(6) (subunit 1, subunit 2)			
$Plane_{equatorial\ F}-Plane_{Py}$	83.82(8)		88.8(1)	
$Plane_{FOF}-Plane_{Py}$	42.04(8) (F1-O1-F3)		56.8(3) (F5-O2-F7)	
	47.90(7) (F2-O1-F4)		32.9(3) (F6-O2-F8)	
$Plane_{equatorial\ F}-Plane_{imidazolium}$	70.31(5) (C2 toward anion)		76.00(8) (C31 toward anion)	
	24.14(7) (C33, C34 toward anion)		20.6(1) (C4, C5 toward anion)	

[a] Bond valence analyses were done using the equation $\sum_j s_{ij} = \sum_j e^{\left(\frac{R^0_{ij} - R_{ij}}{B}\right)}$, with parameters $R_{(V-N)} = 1.86$ Å, $R_{(V-O)} = 1.803$ Å, $R_{(V-F)} = 1.71$ Å, $B = 0.37$ Å.[1, 2]

Table S4. Comparison some of selected structural features in anions $[VOF_4]^-$,[3] $[VOF_4(THF)]^-$ and $[VOF_4(Py)]^-$.

	$[VOF_4]^-$ domain A	$[VOF_4(THF)]^-$	$[VOF_4(Py)]^-$ subunit 1	$[VOF_4(Py)]^-$ subunit 2
Bond length [Å]				
V=O	1.560(2)	1.561(2)	1.570(2)	1.574(2)
V−F1	1.776(2)	1.819(1)	1.840(1)	1.832(1) (F5)
V−F2	1.815(2)	1.805(1)	1.804(1)	1.805(1) (F6)
V−F3	1.807(2)	1.806(1)	1.831(1)	1.821(1) (F7)
V−F4	1.794(2)	1.821(1)	1.811(1)	1.815(1) (F8)
$V-X_{ligand}$[c]	NA[b]	2.403(7) (O30A)<br>2.464(6) (O30B)	2.399(2)	2.406(2)
Cation displacement [Å]				
$V-Plane_F$[c]	0.446	0.309	0.291	0.288
Angle [°]				
O=V−F1	107.0(1)	99.64(7)	99.45(9)	98.62(9) (F5)
O=V−F2	104.5(1)	100.21(9)	99.49(8)	98.07(8) (F6)
O=V−F3	103.1(1)	100.53(7)	99.13(9)	99.34(9) (F7)
O=V−F4	103.4(1)	98.69(8)	98.61(9)	100.40(7) (F8)
$F-V-F_{trans}$	149.8(1) (F1A, F3A)<br>152.08(9) (F2A, F4A)	159.78(6) (F1, F3)<br>161.05(7) (F2, F4)	161.37(6) (F1, F3)<br>161.87(7) (F2, F4)	161.85(7) (F5, F7)<br>161.53(6) (F6, F8)
$O=V-X_{ligand}$	NA[b]	171.3(2) (O30A)<br>175.1(2) (O30B)	179.61(9)	176.78(8)

[a] X represents electron donating moiety of the ligand (O for THF, N for Py).
[b] Not applicable, as the sixth coordination site of the anion is empty.
[c] Distance of V from the plane of F atoms.

Table S5. Selected examples of structurally characterized compounds with vanadium oxofluorido anions.

Compound	V−O	Axial ligand	Bond length of axial ligand [Å]	O−V−O/F_{trans} [°]	Average bond length of equatorial ligands [Å]	Average (range of O−V−F angles [°])	Structural unit	Displacement [Å] Reported	Displacement [Å] Calculated	Reference
$[enH_2][VOF_5]$[a]	1.54(1)	F	2.10(1)	177.6(5)	1.80 (1.75(1)–1.85(1))	98.2 (96.1(5)–101.0(6))	Monomer	0.27	0.258	[4]
$Na_2[VOF_5]$	1.607(6)	F	2.140(6)	180calc	1.892 (1.892(3))	98.1 (98.1(2))	Monomer	0.27	0.266	[5]
$[PyH]_2[Cu(Py)_4(VOF_5)_2]$[b]	1.596(2)	F	2.084(2)	178.27(8)calc	1.837 (1.811(2)–1.883(2))	96.57 (95.71(8)–97.20(9))	Monomer		0.211	[6]
$[4,4'\text{-}bpyH_2][VOF_5]$[c]	1.587(3)	F	2.193(2)	179.6(1)	1.819 (1.802(2)–1.826(2))	97.9 (96.7(1)–98.9(1))	Monomer		0.250	[7]
$Pb[VOF_5]$	1.52(1)	F	2.101(8)	177.1(5)	1.839 (1.838(6)–1.840(7))	98.2 (96.5(3)–99.9(4))	Monomer		0.259	[8]
$[enH_2][VOF_4(H_2O)]$[a]	1.577(3)	H_2O	2.333(2)	177.3(1)	1.808 (1.769(2)–1.858(2))	99.1 (98.0(1)–99.7(1)	Monomer	0.33	0.286	[9]
$[pipH_2]_3[V_2O_2F_8][VOF_4(H_2O)]_2$[d]	1.578(3)	H_2O	2.274(3)	178.0(1)	1.828 (1.795(2)–1.843(2))	99.0 (98.1(1)–100.7(1))	Monomer		0.286	[10]
$Cs[NMe_4][V_2O_2F_8(H_2O)]$[e]	1.581(4)	F	2.322(3)	179.4(2)	1.815 (1.813(3)–1.816(3))	99.9 (99.8(1)–100.0(1))	Corner-shared dimer		0.314	[11]
	1.575(4)	H_2O	2.294(4)	179.0(2)	1.822 (1.804(2)–1.864(3))	98.5 (96.5(2)–100.4(1))			0.321	
$Cs[VOF_4]$	1.53(1)	F	2.31(1)	178.8(7)	1.787 (1.78(1)–1.79(1))	100.4 (98.1(7)–102.0(7))	1D chain	0.326	0.325	[12]
$K[VOF_4]$	1.572(7)	F	2.333(6)	178.4(5)°	1.814 (1.786(7)–1.875(6))	100.0 (98.4(5)–100.9(5))	1D chain	0.31	0.315	
$[EMIm][VOF_4]$[f]	1.575(1)	F	2.3786(7)	173.15(5)	1.8203 (1.7883(8)–1.8633(7))	100.74 (98.52(4)–103.64(5))	Edge-shared dimer		0.333	[13]
$Pb[V_2O_2F_8]$	1.564(5)	F	2.401(4)	175.4(2)	1.830 (1.804(4)–1.871(4))	102.2 (100.3(2)–104.4(2))	Edge-shared dimer		0.386	[8]
$Pb_3F[V_4O_3F_{18}]$	1.55(2)	F	2.38(2)	174(1)	1.84 (1.77(2)–1.88(2))	100.4calculated (97.4–103.0)calculated	Corner-shared tetramer		0.330	[8]
Ag[VOF4]	1.571(3)	F	2.287(2)	179.2(1)	1.824 (1.798(2)–1.857(2))	100.1 (99.3(1)–101.4(1))	1D chain		0.320	[14]
$[NH_3OH][VOF_4]$	1.566(1)	F	2.3306(7)	178.84(4)	1.8262 (1.8094(7)–1.8540(8))	99.96 (99.20(4)–101.07(4))	1D chain		0.316	[15]

[a] en = ethane-1,2-diamine, [b] Py = pyridine, [c] 4,4'-bpy = 4,4'-bipyridine, [d] pip = piperazine, [e] NMe_4 = *N*,*N*,*N*-trimethylmethanaminium, [f] EMIm = 1-ethyl-3-methyl-*1H*-imidazol-3-ium

S2. Comparison of experimental and DFT calculation data

Table S6. Comparison between the PBE-D3/plane-wave calculated and the experimentally determined lattice parameters and unit-cell volumes of [(L^{Dipp})H][VOF_4(THF)] (**1**), [(L^{Dipp})H][VOF_4(Py)] (**2**), and [L^{PPh3Me}][$NbOCl_4$(CH_3CN)] crystal structures. The calculated parameters for the hypothetical [(L^{Dipp})H][VOF_4(H_2O)] and hypothetical [L^{PPh3Me}][$VOCl_4$(CH_3CN)] crystal structures are also reported, which were obtained by replacing Nb with V in [L^{PPh3Me}][$NbOCl_4$(CH_3CN)] and by replacing THF with H_2O in [(L^{Dipp})H][VOF_4(THF)] (**1**) and reoptimizing the crystal structures with the variable-cell relaxations.

	[(L^{Dipp})H] [VOF_4(THF)] (**1**)		[(L^{Dipp})H] [VOF_4(Py)] (**2**)		[(L^{Dipp})H] [VOF_4(H_2O)]		[L^{PPh3Me}] [$VOCl_4$(CH_3CN)]		[L^{PPh3Me}] [$NbOCl_4$(CH_3CN)]	
	PBE-D3	Expt.[a]	PBE-D3	Expt.	PBE-D3	Expt.	PBE-D3	Expt.	PBE-D3	Expt.[c]
a [Å]	12.63	12.66	12.52	12.57	12.61	/	11.40	/	11.55	11.74
b [Å]	12.66	12.78	12.65	12.72	12.73	/	11.13	/	11.24	11.30
c [Å]	19.56	19.61	20.57	20.56	19.33	/	9.38	/	9.42	9.47
α [°]	90.0	90.0	88.7	88.9	90.0	/	97.8	/	97.8	97.7
β [°]	91.9	93.4	80.8	81.2	90.0	/	94.0	/	93.7	93.6
γ [°]	90.0	90.0	87.1	87.1	90.0	/	79.0	/	78.6	78.6
V [Å^3]	3127	3165	3210	3244	3103	/	1157	/	1187	1219

[a] Disordered.

[b] No experimental data available.

[c] Reference[16].

S3. MOPDOS of the $[VOF_4(THF)]^-$ and $[VOF_4(Py)]^-$ complexes: projections to the VOF_4^- anion

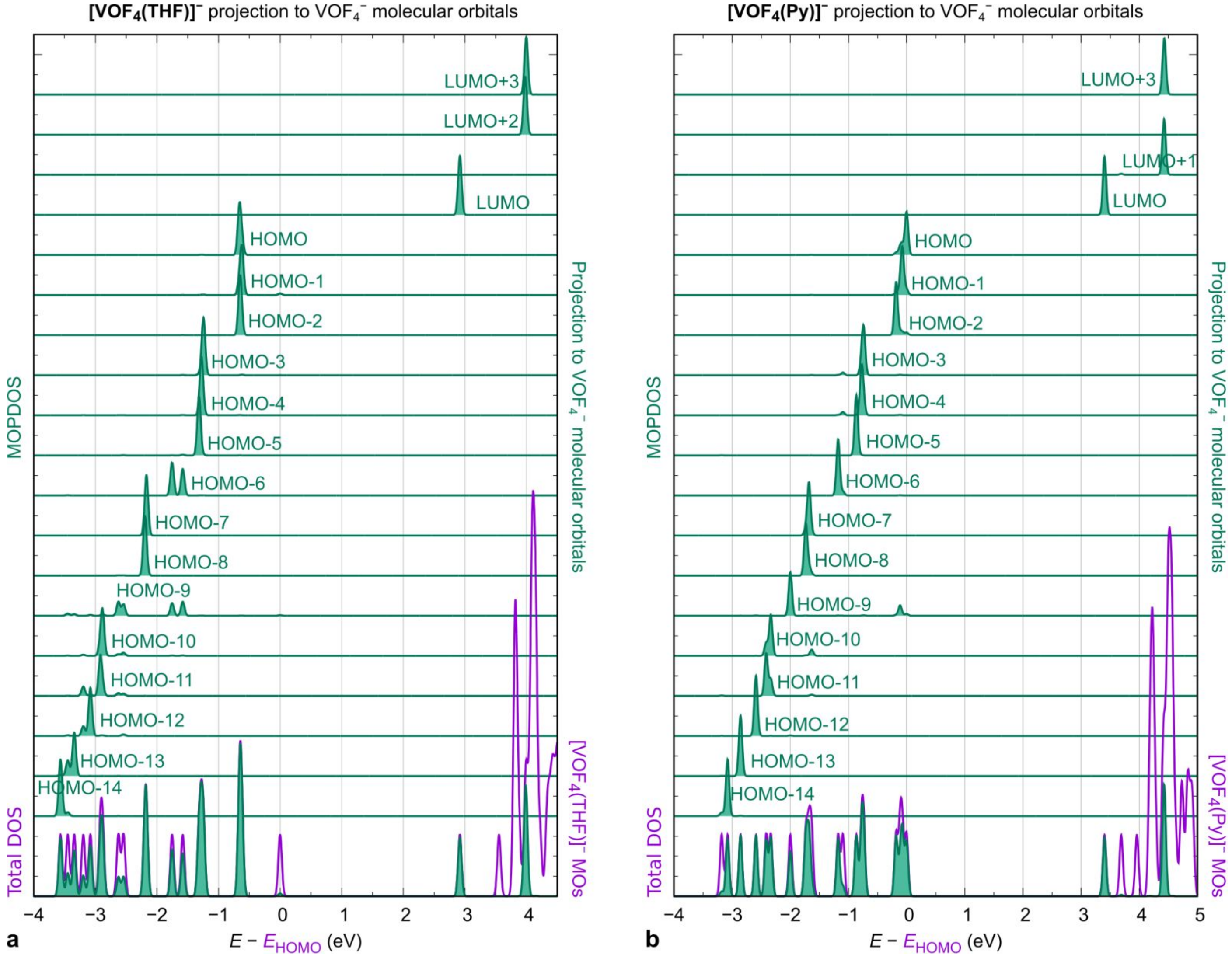

Figure S1: Density of states (DOS) analyses of the standalone (a) $[VOF_4(THF)]^-$ and (b) $[VOF_4(Py)]^-$ complexes. The purple curves at the bottom of the plots represent the total DOS, whereas the superposed green curves are the DOS projections to the VOF_4^- fragment. Above these curves the DOS is projected to individual molecular orbitals of the VOF_4^- anion. The labels HOMO-*n* and LUMO+*m* stand for the *n*th orbital below HOMO and *m*th orbital above LUMO, respectively.

S4. Molecular orbitals of the VOF_4^- anion, Py ligand, and $[VOF_4(Py)]^-$ complex

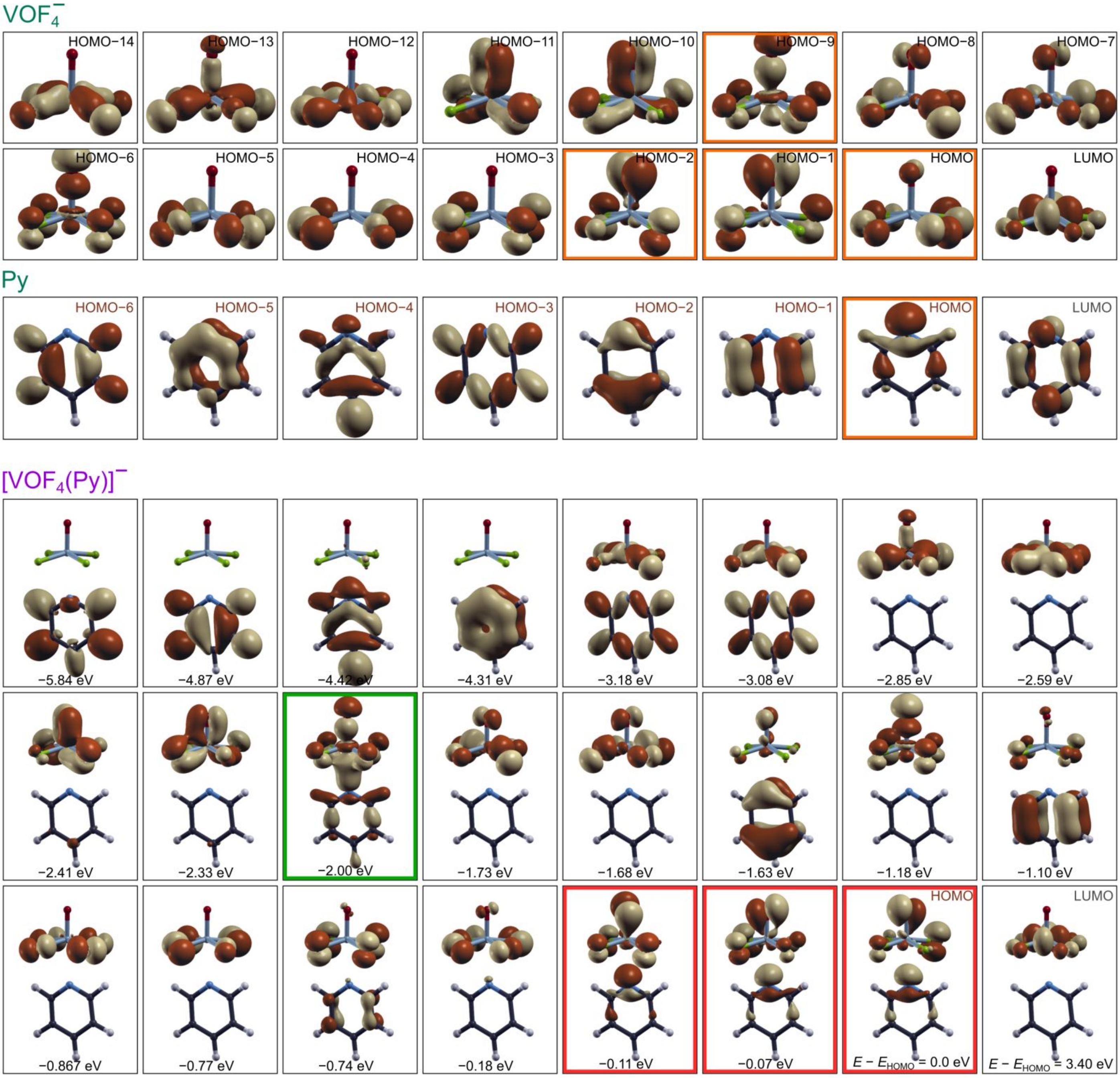

Figure S2: A subset of PBE-D3 calculated signed molecular orbital densities, $\mathrm{sgn}(\psi_i(\mathbf{r}))|\psi_i(\mathbf{r})|^2$, of the VOF_4^- anion, the Py ligand, and the standalone $[VOF_4(Py)]^-$ complex. The label HOMO-*n* stands for the *n*th orbital below HOMO. There is only one bonding molecular orbital between VOF_4 and Py, which is highlighted with green rectangle, whereas molecular orbitals between the two fragments that display more anti-bonding character are highlighted by red rectangles. Molecular orbitals of the individual VOF_4^- and Py fragments that are predominantly involved in the highlighted states are marked with orange rectangles.

S5. Some electronic parameters of ligands and metal centers

Vertical ionization potentials (*I*) and vertical electron affinities (*A*) were calculated as:

$$I = [E_{X^+} - E_X]_\nu \text{ and } A = [E_X - E_{X^-}]_\nu,$$

where *X* stands for the neutral molecule and X^+ and X^- are the corresponding cation and anion, respectively, that correspond to the $X \rightarrow X^+ + e^-$ and $X + e^- \rightarrow X^-$ reactions. To ensure constant external potential ν, the molecular geometries of X^+ and X^- were kept fixed to that of X. The Mulliken electronegativities (χ) and chemical hardnesses (η) were calculated from *I* and *A* as:

$$\chi = \frac{I + A}{2} \text{ and } \eta = \frac{I - A}{2}.$$

The PBE-D3/def2TZVP calculated values of *I*, *A*, χ, and η of the considered ligands are given in Table S7.

Table S7. PBE-D3/def2TZVP calculated vertical ionization potential (*I*), vertical electron affinity (*A*), Mulliken electronegativity (χ), and chemical hardness (η) of the considered ligands.

	I (eV)	A (eV)	χ (eV)	η (eV)
N-ligands				
CH_3CN	12.05	−2.12	4.97	7.09
NH_3	10.89	−2.51	4.19	6.70
Py	9.31	−1.03	4.14	5.17
ABCO	7.64	−1.84	2.90	4.74
O-ligands				
H_2O	12.65	−2.52	5.06	7.58
$OCCH_2$	9.72	−1.39	4.16	5.56
THF	9.38	−2.31	3.54	5.84

The ionization potential and electron affinity and, in turn, electronegativity and chemical hardness of the V(+5) and Nb(+5) metal centers were roughly estimated by two different approaches—note that here trends and not specific values are of interest. In the first approach, the formal oxidation state of +5 is equated with the actual charge of 5+ (this approach is named as "M(+5) = M^{5+} approximation"). In this case, the ionization potential of V^{5+} is given by the sixth ionization potential of V that corresponds to the $V^{5+} \rightarrow V^{6+} + e^-$ reaction, whereas the electron affinity of V^{5+} corresponds to $V^{4+} \rightarrow V^{5+} + e^-$ and is therefore given by the fifth ionization potential of V. In the second approach, the calculated Bader charge of V(5+) center of VOF_4^- is utilized, which equals 2.3. This charge best corresponds to V^{2+} ion, hence the corresponding *I* and *A* values are approximated by the third and second ionization potential of V, respectively (this approach is named as "M(+5) = M^{2+} approximation"). The case of Nb(5+) is treated analogously. The experimental values for the first six ionization potentials of V and Nb are tabulated in Table S8, whereas the *I*, *A*, χ, and η values of V(+5)

and Nb(+5), deduced from the two approximations, are given in Table S9. It can be seen that both approaches univocally show that V(+5) is more electronegative and chemically harder than Nb(5+) thus suggesting that V(+5) is a harder Lewis acid than Nb(+5).

Table S8. Experimentally determined first six ionization potentials of V and Nb, Data taken from ref.[17]

	Ionization potentials (eV)					
	1st	2nd	3rd	4th	5th	6th
V	6.7	14.7	29.3	46.7	65.3	128.1
Nb	6.8	14.3	25.0	38.3	50.5	102.1

Table S9. Approximate vertical ionization potential (*I*), vertical electron affinity (*A*), Mulliken electronegativity (χ), and chemical hardness (η) of V(+5) and Nb(+5) metal centers estimated with aid of the "V(+5) = V^{5+}" and "V(+5) = V^{2+}" approximations and the experimental values for the ionization potentials of V and Nb, taken from Table S8.

	I (eV)	A (eV)	χ (eV)	η (eV)
V(+5) = V^{5+} approximation				
	$I = I^{6th}$	$A = I^{5th}$		
V^{5+}	128.1	65.3	96.7	31.4
Nb^{5+}	102.1	50.5	76.3	25.8
V(+5) = V^{2+} approximation				
	$I = I^{3rd}$	$A = I^{2nd}$		
V^{2+}	29.3	14.7	22.0	7.3
Nb^{2+}	25.0	14.3	19.7	5.4

S6. Estimation of the X···H−C bond strengths

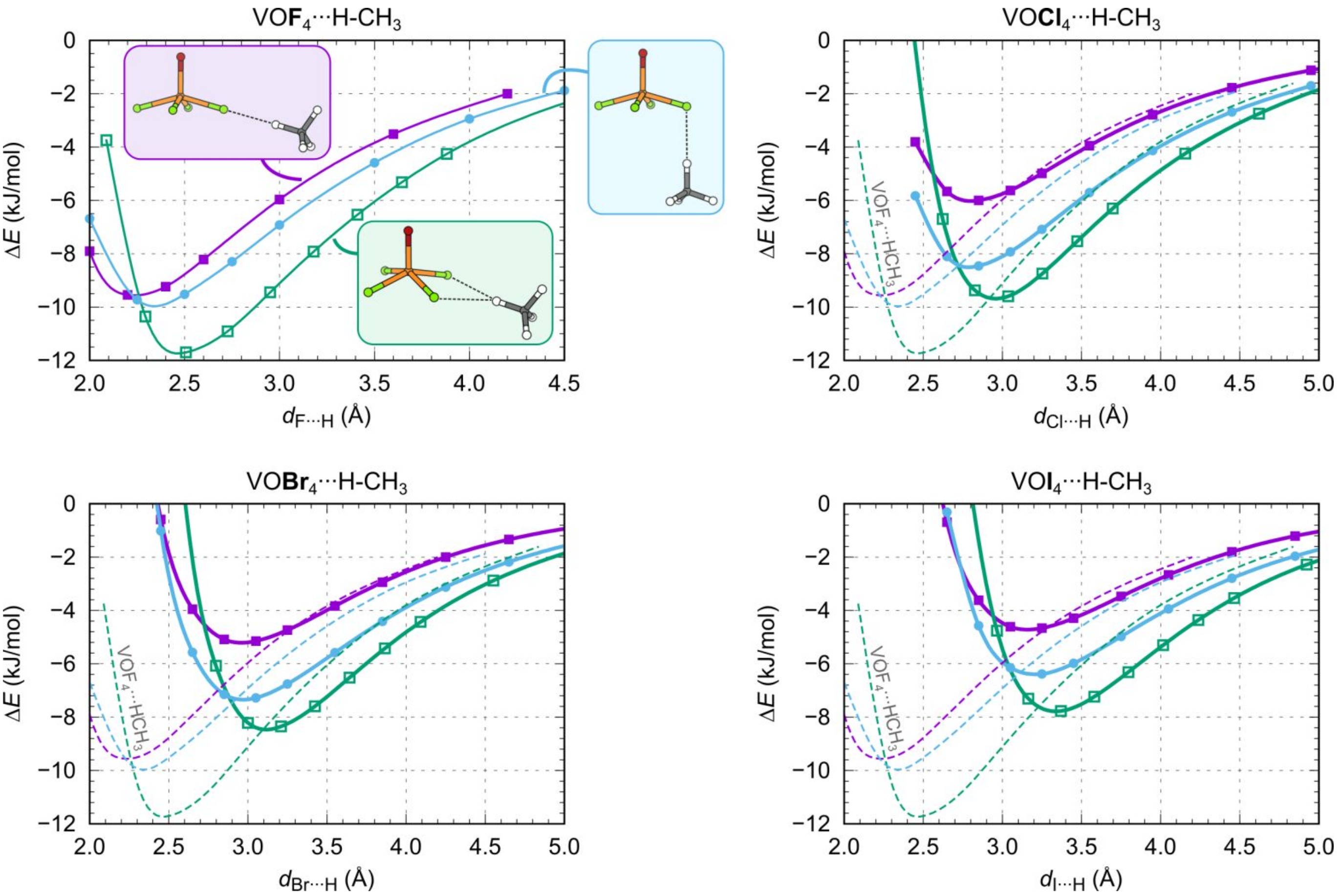

Figure S3. PBE-D3/def2TZVP calculated interaction energies between methane (CH_4) and $[VOX_4]^-$ anions (X = F, Cl, Br, and I) in various geometries as a function of the F···H distance. These calculations were used to estimate the strength of the linear and bifurcated X···H−C bonds.

S7. References

[1] Brown I. D.; Altermatt D.; Bond-Valence Parameters Obtained from a Systematic Analysis of the Inorganic Crystal Structure Database, *Acta Crystallogr. Sect. B* **1985**, *41*(4), 244–247.
[2] Brese N. E.; O'Keeffe M.; Bond-Valence Parameters for Solids, *Acta Crystallogr. Sect. B*, **1991**, *47*(2), pp. 192–197.
[3] Zupanek Ž.; Tramšek M.; Kokalj A.; Tavčar G.; Reactivity of VOF_3 with N-Heterocyclic Carbene and Imidazolium Fluoride: Analysis of Ligand–VOF_3 Bonding with Evidence of a Minute π Back-Donation of Fluoride. *Inorg. Chem.* **2018**, *57* (21), 13866–13879.
[4] Rieskamp H.; Mattes R.; Fluorreiche Oxofluorovanadate(V): Die Kristallstruktur von $[enH_2][VOF_5]$, *Zeitschrift für Naturforsch. B*, **1976**, *31* (11), 1453–1455.
[5] Stomberg R.; Crystal Structure of Sodium Pentafluorooxovanadate(V), $Na_2[VF_5O]$, and Potassium catena-mu-Fluoro-difluorodioxovanadate(V), $K_{2n}[(VF_3O_2)_n]$, Two Decomposition Products in the System $V_2O_5/(MF,HF)/H_2O_2/H_2O$, *Acta Chem. Scand.*, **1986**, *40A*, 325–330.
[6] Welk M. E.; Norquist A. J.; Stern C. L.; Poeppelmeier K. R.; The Structure-Directing Properties of $[VOF_5]^{2-}$, *Inorg. Chem.* **2000,** *39* (18), 3946–3947.
[7] Gautier R.; Chang K. B.; Poeppelmeier K. R.; On the Origin of the Differences in Structure Directing Properties of Polar Metal Oxyfluoride $[MO_x F_{6-x}]^{2-}$ (x = 1, 2) Building Units, *Inorg. Chem.*, **2015**, *54*, (4), 1712–1719.
[8] Lozinšek M.; Goreshnik E.; Žemva B.; Lead Fluoridooxidovanadates(V), $Pb(V_2O_2F_8)$, $Pb(VOF_5)$, and Mixed Valent Fluoridooxidovanadate(IV,V), $Pb_3F(V_4O_3F_{18})$, *Zeitschrift für Anorg. und Allg. Chemie*, **2012**, *638* (12–13), 2123–2128.
[9] Rieskamp H.; Mattes R.; Oxotetrafluoro-vanadate(V): Die Kristallstruktur von $[enH_2][VOF_4(H_2O)]_2$, *Zeitschrift für Naturforsch. B* **1976**, *31*(5), 541–543.
[10] Aldous D. W.; Stephens N. F.; Lightfoot P.; The role of temperature in the solvothermal synthesis of hybrid vanadium oxyfluorides, *Dalt. Trans.* **2007** (37), 4207–4213.
[11] Hilbers M.; Leimkühler M.; Mattes R.; Zwei-und dreikernige Fluorooxoanionen des Vanadins. Kristallstrukturen von $Cs(NMe_4)[V_2O_2F_8(H_2O)]$ und $(Na,K)(NMe_4)_2[V_3O_3F_{12}]$, *Zeitschrift für Naturforsch. B* **1989**, *44* (4), 383–388.
[12] Bushnell G. W.; Moss K. C.; The Crystal Structure of Caesium Oxotetrafluorovanadate(V), *Can. J. Chem.* **1972**, *50* (22), 3700–3705.
[13] Kanatani T.; Matsumoto K.; Hagiwara R.; Syntheses and Physicochemical Properties of Low-Melting Salts Based on VOF_4^- and $MoOF_5^-$, and the Molecular Geometries of the Dimeric $(VOF_4^-)_2$ and $Mo_2O_4F_6^{2-}$ Anions, *Eur. J. Inorg. Chem.*, **2010**, *2010* (7), 1049–1055.
[14] Lozinšek M.; Goreshnik E.; Žemva B.; Silver(I) Tetrafluoridooxidovanadate(V) – $Ag[VOF_4]$, *Acta Chim. Slov.* **2014,** *61* (3), 542–547.
[15] Lozinšek M.; Hydroxylammonium Tetrafluoridooxidovanadate(V) – $(NH_3OH)[VOF_4]$, *Acta Chim. Slov.*, **2015,** *62* (2) 378–384.
[16] Hiller W.; Strähle J.; Prinz H.; Dehnicke K.; Die Kristallstruktur von $PPh_3Me[NbOCl_4(CH_3CN)]$. *Zeitschrift für Naturforsch. B* **1984**, *39* (1), 107–110.
[17] Lide D. R. (editor-in-chief) (2003–2004). "Section 10. Atomic, Molecular, and Optical Physics". Ionization Potentials of Atoms and Atomic Ions. CRC Handbook of Chemistry and Physics (84th edition). CRC Press. pp. 10-178 to 10-180.
